# *Ppp6c* deficiency accelerates *K‐ras* *^G12D^*‐induced tongue carcinogenesis

**DOI:** 10.1002/cam4.3962

**Published:** 2021-06-18

**Authors:** Kazuhiro Kishimoto, Kosuke Kanazawa, Miyuki Nomura, Takuji Tanaka, Taeko Shigemoto‐Kuroda, Katsuya Fukui, Koh Miura, Koreyuki Kurosawa, Masaaki Kawai, Hiroyuki Kato, Keiko Terasaki, Yoshimi Sakamoto, Yoji Yamashita, Ikuro Sato, Nobuhiro Tanuma, Keiichi Tamai, Issay Kitabayashi, Kazuto Matsuura, Toshio Watanabe, Jun Yasuda, Hiroyuki Tsuji, Hiroshi Shima

**Affiliations:** ^1^ Division of Cancer Chemotherapy Miyagi Cancer Center Research Institute Miyagi Japan; ^2^ Division of Cancer Molecular Biology Tohoku University School of Medicine Miyagi Japan; ^3^ Department of Head and Neck Surgery Kanazawa Medical University Kanazawa Japan; ^4^ Department of Head and Neck Surgery Miyagi Cancer Center Miyagi Japan; ^5^ Division of Surgery Miyagi Cancer Center Miyagi Japan; ^6^ Research Center of Diagnostic Pathology Gifu Municipal Hospital Gifu Japan; ^7^ Department of Plastic and Reconstructive Surgery Tohoku University School of Medicine Miyagi Japan; ^8^ Division of Pathology Miyagi Cancer Center Miyagi Japan; ^9^ Division of Cancer Stem Cell Miyagi Cancer Center Research Institute Miyagi Japan; ^10^ Division of Hematological Malignancy National Cancer Center Research Institute Tokyo Japan; ^11^ Department of Head and Neck Surgery National Cancer Center Hospital East Chiba Japan; ^12^ Department of Biological Science, Graduate School of Humanities and Sciences Nara Women’s University Nara Japan; ^13^ Division of Molecular and Cellular Oncology Miyagi Cancer Center Research Institute Miyagi Japan

**Keywords:** chemokines, head and neck squamous cell carcinoma, K‐ras, protein phosphatase 6, Trp53

## Abstract

**Background:**

Effective treatments for cancer harboring mutant RAS are lacking. In Drosophila, it was reported that PP6 suppresses tumorigenicity of mutant RAS. However, the information how PP6 regulates oncogenic RAS in mammals is limited.

**Methods:**

We examined the effects of PP6 gene (Ppp6c) deficiency on tongue tumor development in K (K‐rasG12D)‐ and KP (K‐rasG12D + Trp53‐deficient)‐inducible mice.

**Results:**

Mice of K and KP genotypes developed squamous cell carcinoma in situ in the tongue approximately 2 weeks after the induction of Ppp6c deficiency and was euthanized due to 20% loss of body weight. Transcriptome analysis revealed significantly different gene expressions between tissues of Ppp6c‐deficient tongues and those of Ppp6c wild type, while Trp53 deficiency had a relatively smaller effect. We then analyzed genes commonly altered by Ppp6c deficiency, with or without Trp53 deficiency, and identified a group concentrated in KEGG database pathways defined as ‘Pathways in Cancer’ and ‘Cytokine‐cytokine receptor interaction’. We then evaluated signals downstream of oncogenic RAS and those regulated by PP6 substrates and found that in the presence of K‐rasG12D, Ppp6c deletion enhanced the activation of the ERK‐ELK1‐FOS, AKT‐4EBP1, and AKT‐FOXO‐CyclinD1 axes. Ppp6c deletion combined with K‐rasG12D also enhanced DNA double‐strand break (DSB) accumulation and activated NFκB signaling, upregulating IL‐1β, COX2, and TNF.

## Graphical abstract

Here, we examined the effects of PP6 gene (Ppp6c) deficiency on tongue tumor development in K (K‐rasG12D)‐ and KP (K‐rasG12D + Trp53‐deficient)‐inducible mice. Mice of K and KP genotypes developed squamous cell carcinoma in situ in the tongue approximately 2 weeks after the induction of Ppp6c deficiency. Ppp6c deletion in the presence of K‐rasG12D enhanced the activation of the ERK‐Elk1‐FOS, AKT‐4EBP1, and AKT‐FOXO‐CyclinD1 axes. Ppp6c deletion combined with K‐rasG12D also activated NFkB signaling, upregulating TNF which leads to an explosion of cytokines. We found that PP6 functions as a tumor suppressor by suppressing the activity of ERK, AKT, and NFkB.

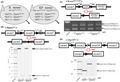

## 1 INTRODUCTION

Head and neck squamous cell carcinoma (HNSCC) has a yearly incidence of 60,000 cases worldwide, with 40%–50% mortality.^1^, ^2^ In HNSCCs that are HPV (−), *EGFR*/*ERBB*2, or *EGFR*1/3 alterations were most frequent among receptor tyrosine kinases. Kinase targets, such as *HRAS*, *PI3CA*, and *PTEN*, also showed mutations implying perturbed RTK/RAS/PI3K signaling in HNSCC carcinogenesis.^1^, ^2^ Moreover, the tumor suppressor *Trp53* was mutant in 84% of HNSCCs that were HPV (−).^1^, ^2^

Currently, effective treatments for cancers harboring mutations in RAS family genes (*HRAS*, *NRAS*, and *KRAS*) are lacking,^3^ partly because of an incomplete understanding of signaling within those tumors. A recent large‐scale ethyl methanesulfonate (EMS)‐induced genetic screen in *Drosophila* identified loss of protein phosphatase 6 (PP6) as cooperating with oncogenic Ras to induce tumor cell proliferation and invasion,^4^ suggesting that PP6 serves as a tumor‐suppressor in RAS‐related cancers. PP6 is a member of Ser/Thr protein phosphatases that binds to any one of the three regulatory proteins PP6R1, PP6R2, and PP6R3, which confer substrate specificity.^5^ Diverse phenotypes seen following from siRNA‐based *Ppp6c* knockdown in cultured mammalian cells suggest that PP6 regulates mitosis by dephosphorylating Aurora kinase A,^6^ activates DNA‐PK to sensitize cells to ionizing radiation,^7^ and is required for homology‐directed repair.^8^ There is also evidence that PP6 regulates NFκB signaling by blocking IκBε degradation in response to TNF^9^ and inactivating TAK1.^10^

Accumulating pathological evidence suggests that *Ppp6c* may function as a tumor suppressor. The PP6 gene (*Ppp6c*) is reportedly mutated in 12% of human melanoma tissues^11^ and 15% of human skin basal cell carcinoma tissues,^12^ and PP6 expression is known to be repressed in some solid tumors. Some human breast cancers also show decreased protein levels of PP6, PP6R1, PP6R2, and PP6R3.^8^ Furthermore, there is evidence that *Ppp6c* is epigenetically regulated. For example, in psoriasis patients, *miR*‐*31*,^13^ which is transcriptionally enhanced by NFκB, suppresses *Ppp6c* expression and promotes abnormal epidermal cell proliferation. Hepatocellular carcinoma cells also reportedly undergo hyperproliferation following *Ppp6c* suppression by upregulated *miR*‐*373* expression.^14^

We previously assessed *Ppp6c* function in a mouse model of skin carcinogenesis and found that *Ppp6c* loss in keratinocytes promoted 7,12‐dimethylbenz[a]anthracene (DMBA)‐induced papilloma formation^15^ and UVB‐induced carcinogenesis.^16^ These findings support the idea that *Ppp6c* acts as a tumor suppressor in mouse skin cancers. Here, we analyzed PP6 function in HNSCCs by assessing mouse tongue carcinogenesis. To do so, we asked whether *Ppp6c* deficiency enhanced tongue carcinogenesis in *K*‐*ras^G12D^* and *Trp53*‐null mouse models.

## 2 MATERIALS AND METHODS

### 2.1 Generation of mice with inducible Kras^G12D^ expression, Trp53 deletion, and Ppp6c deletion

The mouse strain *Ppp6c^flox^* ^/^ *^flox^* has been described.^17^ *ROSA26*‐*CreER^T2^* mice^18^ were obtained from Taconic Bioscience. *K*‐*ras^LSL^* ^‐^ *^G12D^* ^/+^ mice^19^ and *Trp53^flox^* ^/^ *^flox^* mice^20^ were obtained from the Jackson Laboratory.

**FIGURE 1:**
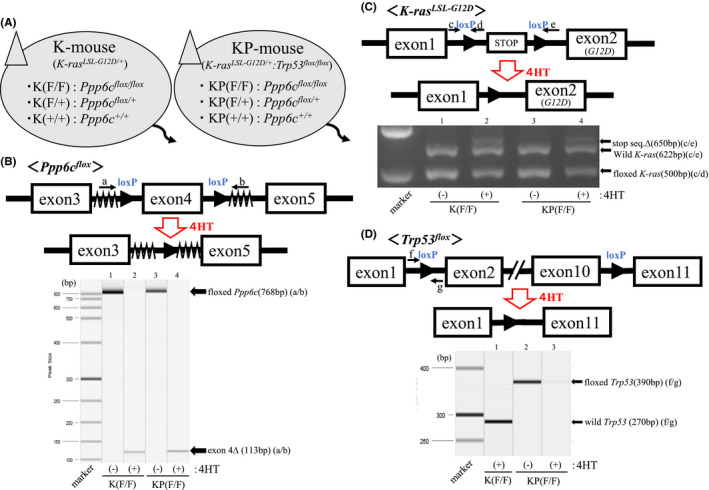
*Ppp6c* deficiency promotes significant tongue thickening and early death in K and KP mice. Genotypes of K and KP mice and *CreER^T2^*‐mediated gene recombination. (A) Genotypes of K‐mouse (left) and KP‐mouse (right). The symbols K(+/+), K(F/+), and K (F/F) denote the genotypes *ROSA26*‐*CreER^T2^*/*K*‐*ras^LSL^* ^‐^ *^G12D^* ^/+^/*Ppp6c* ^+/+^, *ROSA26*‐*CreER^T2^*/*K*‐*ras^LSL^* ^‐^ *^G12D^* ^/+^/*Ppp6c^flox^* ^/+^, and *ROSA26*‐*CreER^T2^*/*K*‐*ras^LSL^* ^‐^ *^G12D^* ^/+^/*Ppp6c^flox^* ^/^ *^flox^*, respectively. (right) KP symbols are the same but with *Trp53^flox^* ^/^ *^flox^*. (B) *CreER^T2^*‐mediated *Ppp6c* disruption. (top) Schematic representation of *Ppp6c*‐floxed and deletion of exon 4 following CreER^T2^ activation. Positions of primers A, and B are indicated., *loxP* sequence. Serrated regions, vector sequence. (bottom) PCR analysis of recombined allele. Lanes 1&2: K(F/F) mice; lanes 3&4: KP(F/F) mice. Lanes 2&4: genomic DNA from the tongue of mice treated with 4HT for 7 days. Lanes 1&3: genomic DNA from the tail of corresponding mice before 4HT treatment. Fragments of 768 and 113 bp correspond to floxed and exon four‐deleted alleles, respectively. (C) *CreER^T2^*‐mediated *Kras^G12D^* expression. (top) Schematic representation of a K‐ras^LSL‐G12D^ allele and deletion of the transcription stop sequence by activated CreER^T2^. (bottom) PCR analysis of recombined allele. Lanes 1&2: K(F/F) mice; lanes 3&4: KP(F/F) mice. Lanes 2&4: genomic DNA obtained from the tongue of mice treated 7 days with 4HT. Lanes 1&3: genomic DNA from the tail of corresponding mice before 4HT treatment. Fragments of 650, 622, and 500 bp correspond to the stop sequence‐deleted allele (amplified with primers C and E), wild‐type allele (amplified with primers C and E), and floxed alleles (amplified with primers C and D), respectively. (D) *CreER^T2^*‐mediated *Trp53* disruption. (top) Schematic representation of *p53*‐floxed allele and deletion of exons 2–10 by activated CreER^T2^. Positions of primers f and g are indicated. (lower) PCR analysis of recombined allele. Lane 1: K(F/F) mice; lanes 2&3: KP(F/F) mice. Lanes 1&3: genomic DNA obtained from tongue mice treated 7 days with 4HT. Lane 2: genomic DNA from the tail of corresponding mice before 4HT treatment. Fragments of 390 and 270 bp correspond to floxed and wild alleles, respectively.

*ROSA26*‐*CreER^T2^* mice were crossed with *K*‐*ras^LSL^* ^‐^ *^G12D^* ^/+^ mice to generate *ROSA26*‐*CreER^T2^*/*K*‐*ras^LSL^* ^‐^ *^G12D^* ^/+^ mice, which were then bred with *Ppp6c^flox^* ^/^ *^flox^* mice to generate *ROSA26*‐*CreER^T2^*/*K*‐*ras^LSL^* ^‐^ *^G12D^* ^/+^/*Ppp6c^flox^* ^/+^ mice. These mice were further crossed with *Ppp6c^flox^* ^/+^ to generate mice of the following three genotypes: *ROSA26*‐*CreER^T2^*/*K*‐*ras^LSL^* ^‐^ *^G12D^* ^/+^ *Ppp6c^flox^* ^/^ *^flox^* (designated K[F/F] mice), *ROSA26*‐*CreER^T2^*/*K*‐*ras^LSL^* ^‐^ *^G12D^* ^/+^/*Ppp6c^flox^* ^/+^ (designated K[F/+] mice), and *ROSA26*‐*CreER^T2^*/*K*‐*ras^LSL^* ^‐^ *^G12D^* ^/+^/*Ppp6c* ^+/+^(designated K[+/+] mice) (Figure 1A left).

We also crossed *ROSA26*‐*CreER^T2^*/*K*‐*ras^LSL^* ^‐^ *^G12D^* ^/+^/*Ppp6c^flox^* ^/+^ mice with *Trp53^flox^* ^/^ *^flox^* mice to obtain *ROSA26*‐*CreER^T2^*/*K*‐*ras^LSL^* ^‐^ *^G12D^* ^/+^/*Trp53^flox^* ^/^ *^flox^*/*Ppp6c^flox^* ^/+^ mice. Following the crossing of *ROSA26*‐*CreER^T2^*/*K*‐*ras^LSL^* ^‐^ *^G12D^* ^/+^/*Trp53^flox^* ^/^ *^flox^*/*Ppp6c^flox^* ^/+^ with *Trp53^flox^* ^/^ *^flox^*/*Ppp6c^flox^* ^/+^ mice, we obtained mice of three genotypes: *ROSA26*‐*CreER^T2^*/*K*‐*ras^LSL^* ^‐^ *^G12D^* ^/+^/*Trp53^flox^* ^/^ *^flox^*/*Ppp6c^flox^* ^/^ *^flox^* (designated KP[F/F] mice), *ROSA26*‐*CreER^T2^*/*K*‐*ras^LSL^* ^‐^ *^G12D^* ^/+^/*Trp53^flox^* ^/^ *^flox^*/*Ppp6c^flox^* ^/+^ (designated KP[F/+] mice) and *ROSA26*‐*CreER^T2^*/*K*‐*ras^LSL^* ^‐^ *^G12D^* ^/+^/*Trp53^flox^* ^/^ *^flox^*/*Ppp6c* ^+/+^ (designated KP[+/+] mice) (Figure 1A right). Littermates served as controls. All animal experiments were performed with approval of the Miyagi Cancer Center Research Institute Animal Care and Use committee (MCCAE‐2020‐1).

### 2.2 4‐Hydroxytamoxifen treatment

4‐Hydroxytamoxifen (4HT), purchased from Toronto Research Chemicals, was used to induce CreER^T2^‐dependent recombination, as reported.^18^ Entire upper surface of tongues of 8‐week‐old mice was painted with 4HT at 10 mg/ml in ethanol three times every other day for a week.

### 2.3 PCR genotype analysis

To confirm exon 4 deletion from floxed *Ppp6c*, we used primers: (a) 5′‐TATCACGAGGCCCTTTCG‐3′; (b) 5′‐TAGTGAACCTCTTCGAGG‐3′ (Figure 1B). To confirm excision of the loxP‐flanked transcription stop cassette from the *LSL*‐*K*‐*ras^G12D^* allele, we used primers; (c) 5′‐GTCTTTCCCCAGCACAGTGC‐3′; (d) 5′‐CTCTTGCCTACGCCAGCT‐3′; (e) 5′‐AGCTAGCCACCATGGCTTGAGTAAGTCTGCA‐3′ (Figure 1C). To confirm the deletion of exons 2–10 from the p53‐floxed allele, we used primers; (f) 5′‐GGTTAAACCCAGCTT GACCA‐3′; (g) 5′‐GGAGGCAGAGACAGTTGGAG‐3′ (Figure 1D).

### 2.4 Phosphoprotein microarray analysis

Phospho Explorer Antibody Microarray designed by Moon BioSystems, was used. For more information, see Doc S1.

### 2.5 Histopathology and immunohistochemistry

Histopathology and immunohistochemistry were performed as previously described.^15^ For immunohistochemistry, the following antibodies were used: anti‐cytokeratin 5 (CK5) (#53121), anti‐Ki‐67 (#15580), anti‐p‐Elk1 (#28818) and anti‐p‐Akt (#38449), all from Abcam; and anti‐γH2AX (#9718), anti‐p‐Erk1/2 (#4370), anti‐p‐4EBP1 (#2855), anti‐MCM2 (#3619) and anti‐NFκB p65 (RelA) (#8242), all from Cell Signaling Technology. e‐Count2 software was used to count Ki‐67‐ and MCM2‐positive nuclei. Phosphorylation levels of ERK1/2 and AKT were evaluated by assessing the intensity of immunoreactivity in the cytoplasm on a 0 to 4+ scale. A pathologist (T.T.) determined the presence of SCCIS epithelial lesions based on the location of cancer cells in the whole epithelium but lack of invasivity.^21^

### 2.6 RNA preparation and sequencing

Total RNA was extracted from fresh frozen tissue using RNeasy Plus Universal Mini Kit (QIAGEN). For more information, see Doc S1.

### 2.7 Transcriptome analysis

To evaluate expression levels of differentially expressed genes between groups, transcriptome analysis was performed. For more information, see Doc S1.

### 2.8 Statistical analysis

Kaplan–Meier survival curves and corresponding statistical analysis, as well as log‐rank tests, were performed using Prism version 8 (GraphPad Software Inc.). Other assessment of statistical significance was performed using Student's *t* test. *p* < 0.05 served as the cut‐off for significance.

## 3 RESULTS

### 3.1 Ppp6c deficiency promotes significant tongue thickening and early death in K and KP mice

Starting with 4HT‐inducible KRAS(G12D) expressing mice (*K*‐*ras^LSL^* ^‐^ *^G12D^* ^/+^: K mice), we generated three lines: mice also homozygous for the *Ppp6c^flox^* ^/^ *^flox^* allele, heterozygous for that allele (*Ppp6c^flox^* ^/+^), and wild‐type (*Ppp6c* ^+/+^) mice and designated them K(F/F), K(F/+) and K(+/+), respectively (Figure 1A left). We also generated 4HT‐inducible KRAS(G12D) expressing plus 4HT‐inducible *Trp53* deficient mice (*K*‐*ras^LSL^* ^‐^ *^G12D^* ^/+^/*Trp53^flox^* ^/^ *^flox^*: KP mice), which have the same three *Ppp6c^flox^* genotypes, KP(F/F), KP(F/+) and KP(+/+) (Figure 1A right).

When mice were 8 weeks old, we used a brush to apply 4HT to the entire upper surface of the tongues of K(F/F) and KP(F/F) mice and assessed genomic recombination in tongue tissue a week later. In all groups, we observed the recombination of the *Ppp6c^flox^* (Figure 1B), *K*‐*ras^LSL^* ^‐^ *^G12D^* (Figure 1C), and *Trp53^flox^* (Figure 1D) alleles. Mice of other genotypes, including K(F/+), K(+/+), KP(F/+), and KP(+/+), were similarly treated with 4HT, and we observed *Ppp6c^flox^*, *K*‐*ras^LSL^* ^‐^ *^G12D^*, and *Trp53^flox^* recombination in tongue tissue in all cases (data not shown).

**FIGURE 2:**
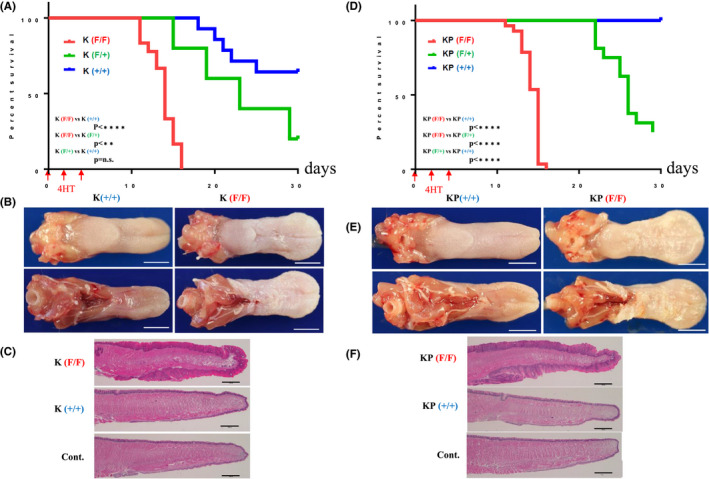
*Ppp6c* deficiency causes significant tongue thickening and early death in K and KP mice. (A–C) Analysis of K mice: (A) K(+/+) (*n* = 13), K(F/+) (*n* = 15) and K(F/F) (*n* = 23) mice were used for this experiment. Tongues of 8‐week‐old mice were painted with 4HT at 10 mg/ml in ethanol three times every other day for a week, and body weight was assessed every day until day 30. When the body weight decreased by 20%, mice were euthanized for pathological analysis. (B) Representative macroscopic views of tongues of 4HT‐treated K(+/+) mouse (left) and 4HT‐treated K(F/F) mouse (right) are shown. Top: ventral surface of tongue. Bottom: dorsal surface of tongue. Scale bar: 3 mm. (C) H&E staining of the whole tongue section. Top: 4HT‐treated K(F/F) tongue shown in (B) right. Middle: 4HT‐treated K(+/+) tongue shown in (B) left. Bottom: tongue from 8‐weeks‐old C57BL/6 mouse. Scale bar: 1 mm. (D–F): Analysis of KP mice: (D) KP(+/+) (*n* = 12), KP(F/+) (*n* = 10) and KP(F/F) (*n* = 29) were used for this experiment. The experiment was performed as described in (A). (E) Representative macroscopic views of tongues of 4HT‐treated KP(+/+) mouse (left) and 4HT‐treated KP(F/F) mouse (right) are shown. Top: front of tongue. Bottom: back of tongue. Scale bar: 3 mm. (F) H&E staining of the whole tongue section. Top: 4HT‐treated KP(F/F) tongue shown in (E), right. Middle: 4HT‐treated KP(+/+) tongue shown in (E), left. Bottom: tongue from 8‐weeks‐old C57BL/6 mouse (control). Scale bar: 1 mm

We then checked body weight every day up to 30 days after the first 4HT application to the tongue (Figure 2) and euthanized mice that had lost 20% of body weight. K(F/F) mice showed a statistically significant weight loss of ~20% at around day 13 after induction and were euthanized (Figure 2A), while the rate of weight loss in K(+/+) relative to K(F/F) mice was slower and differed in a statistically significant manner. Weight loss in K(F/+) mice occurred at a rate midway between that seen in K(F/F) and K(+/+) mice (Figure 2A). By 13 days after 4HT induction, the tongues of K(F/F) mice grossly showed thickening across the ventral and dorsal sites (Figure 2B, Figure S1A). On the H&E stained tongue tissue from mice with *Ppp6c* homozygous deletion in the presence of *K*‐*ras^G12D^*, the entire squamous epithelium was markedly thickened in comparison to that of mice expressing *K*‐*ras^G12D^* alone which was indistinguishable from wild‐type tongues (Figure 2C, Figure S1B).

In KP mice, effects of *Ppp6c* deficiency were similar to those seen in K mice: relative to *Ppp6c* (F/+) and (+/+) KP mice, *Ppp6c* (F/F) KP mice lost weight more rapidly and that difference was statistically significant (Figure 2D). Gross appearance and histopathological findings of the 4HT‐treated KP(F/F) tongue (Figure 2E,F) were comparable to phenotypes seen in K(F/F) mice (Figure 2B,C).

Autopsy analysis performed to determine the cause of weight loss in 4HT‐treated K(F/F) and KP(F/F) mice showed that their stomachs contained less food content than did stomachs of 4HT‐treated K(+/+) and KP(+/+) mice. Macroscopic examination revealed no tumors in the esophagus, stomach, or intestine. These observations suggest that large tongue tumors interfere with feeding, causing mice to become asthenic.

### 3.2 Ppp6c deficiency induces squamous cell carcinoma in situ in tongues of K and KP mice

**FIGURE 3:**
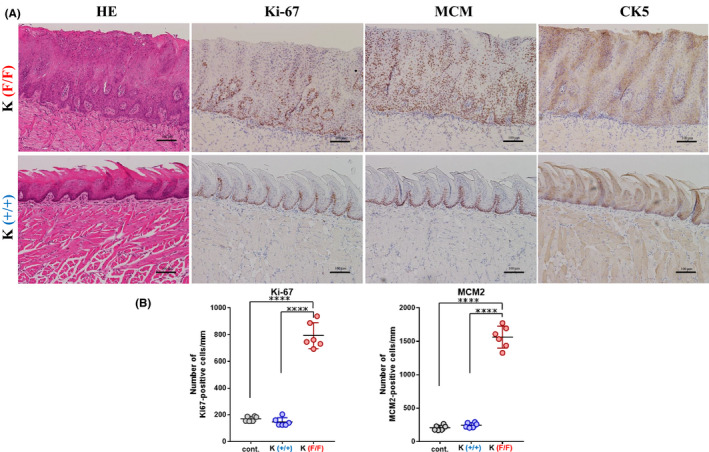
*Ppp6c* deletion induces squamous cell carcinoma in situ in K mice by 2 weeks. (A) Microscopic analysis of sections of 4HT‐treated epithelium from K(F/F) mice and that of from K(+/+) mice shown in Figure 2C. Shown is H&E staining plus immunohistochemistry with anti‐Ki‐67, anti‐MCM2, and anti‐CK5 antibodies. Scale bar: 100 μm. (B) The number of Ki‐67‐positive cells (left) and MCM2‐positive cells (right) in mouse tongue epithelium, counted using samples shown in (A) and from an immunohistogram obtained by staining sections of wild‐type tongue (used in Figure 2C, bottom) with Ki‐67 and MCM2 antibodies. In each sample, Ki67‐positive cells in a ~1 mm wide region of the epidermis were counted. Data are means derived from six areas in independent samples ±SE. ****p < 0.0001

**FIGURE 4:**
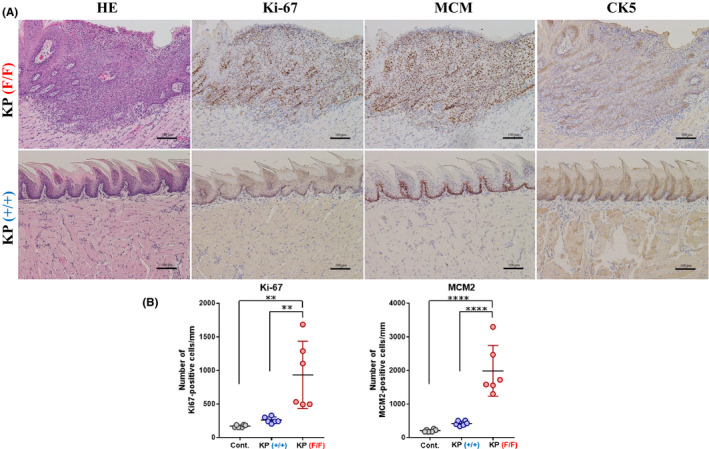
(A) Microscopic analysis of sections of tongue epithelium from 4HT‐treated KP(F/F) mice shown in Figure 2F. Staining was performed as in Figure 3A. (B) The number of Ki‐67‐positive cells and MCM2‐positive cells were obtained as in Figure 3B. Data are means derived from six areas in independent samples ±SE. **p < 0.001. ****p < 0.0001

To assess morphological alterations and growth of tongue epithelial cells, we next performed immunohistochemistry analysis. As shown in Figure 3A, which are high power of views of Figure 2C, tumorous lesions were observed in the 4HT‐treated K(F/F) tongue tissue stained with H&E. In lesions found in 4HT‐treated K(F/F) tongue, atypical cells throughout the epithelium showed loss of cell polarity, although we did not observe stromal invasion of these cells (Figure 3A). Immunohistochemical staining using antibodies, two cell proliferation markers Ki‐67 and MCM2 and the squamous cell marker CK5 on serial sections showed nuclear positive reactivities of Ki‐67 and MCM2 and cytoplasmic positivity of CK5 throughout the tongue epithelium of the 4HT‐treated K(F/F) mice, suggesting squamous cell carcinoma in situ (SCC in situ/SCCIS). In addition, the numbers of Ki‐67 and MCM2‐positive cells of the K(F/F) tongue epithelium were significantly higher than those of the wild‐type and K(+/+) tongues, both being comparable (Figure 3B). Similar tumorous lesions emerged in the 4HT‐treated KP(F/F) tongue (Figure 4) and were also diagnosed as SCCIS. Immunohistochemical findings were seen in the 4HT‐treated KP(F/F) tongue and those of K(F/F) were comparable (Figures 3a and 4a). Mild dysplasia developed in the tongue of F/F mice 1 week after 4HT application, and by 13 days, tongue SCCIS developed in all F/F mice that showed a decrease in body weight of 20% or more. Incidences of thickened squamous epithelium and SCCIS were 100% and 100%, respectively, by 13 days. Various degrees of squamous dysplasia occurred in all thickened squamous epithelium. We observed SCCIS in some areas of dysplastic lesions based on loss of cell polarity, severe nuclear atypia, and abnormal mitoses. These results indicate that *Ppp6c* loss promotes the development of SCCIS in squamous cells expressing *K*‐*ras^G12D^* within 13 days and that *Trp5*3 status has a minimal effect on the process.

**FIGURE 5:**
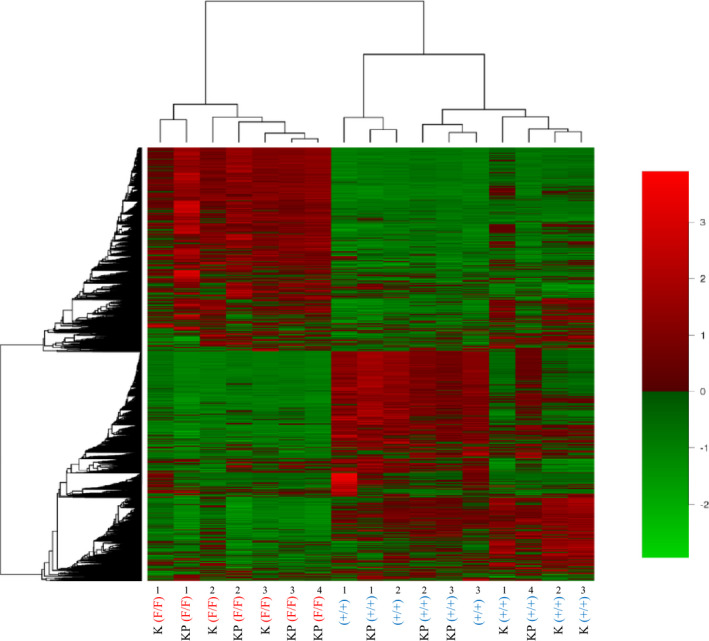
Heat map analysis of gene expression in the 4HT‐treated tongue of K(F/F), K(+/+), KP(F/F), KP(+/+), and control mice. K(F/F) (*n* = 3), K(+/+) (*n* = 3), KP(F/F) (*n* = 4), and KP(+/+) (*n* = 4) mice plus normal (*n* = 3) control C57BL/6 (designated as [+/+]) mice were painted with 4HT at 10 mg/ml in ethanol on the tongue three times every other day for a week, and then 13 days later epithelial tongue tissue was harvested for mRNA preparation and transcriptomic analysis. The heat map was generated as described in Materials and Methods. A total of 15,921 genes expressed in at least one of the groups in each comparison were used for this analysis. The heat map shows differentially expressed mRNAs in K and KP mice with *Ppp6c* deficiency (F/F) or wild‐type (+/+), and control (C57BL/6) mice. Red and green colors indicate high and low expression levels, respectively

Next, we examined gene expression in K(F/F) and KP(F/F) tumors (Figure 5). For these experiments, we treated 3 K(F/F) mice, 3 K(+/+) mice, 4 KP(F/F) mice, 4 KP(+/+) mice, plus three normal control C57BL/6 mice in the same manner described in Figure 1. Figure 5 shows a heat map representing gene expression in the epithelium of tongue tissue from each genotype, indicating that expression patterns of K and KP mice differed significantly between *Ppp6c* wild‐type and ‐deficient mice. Again, the effect of *Trp53* deficiency was relatively small compared to that of *Ppp6c* deficiency.

### 3.3 Identification of signaling pathways activated by Ppp6c deficiency in K and KP mice

To identify signaling pathways underlying SCCIS development over a 2‐week timeframe, we characterized pathways commonly altered by *Ppp6c* loss in both K and KP mice. Table 1 shows signaling pathways altered by *Ppp6c* loss in the presence of *K*‐*ras^G12D^* in both K and KP mice. Those significantly (*p *< 0.05) altered are shown in order of decreasing *p* value in K mice. Of particular interest were ‘Pathways in Cancer’ (ranked 16 in Table 1) and ‘Cytokine‐cytokine receptor interaction’ (ranked 1 in Table 1), as these pathways are linked to RAS and PP6, respectively.

**TABLE 1:** Signally pathways commonly affected by *Ppp6c* deletion in K and KP mice

**Rank**	KEGG‐pathway	K mice	KP mice	# genes (DE/ALL)	*p*‐value
		# genes (DE/ALL)	*p*‐value		
1	Cytokine‐cytokine receptor interaction	68/165	1.12E‐08	93/169	1.05E‐06
2	Protein digestion and absorption	32/58	2.09E‐08	40/58	1.30E‐05
3	ECM‐receptor interaction	34/66	1.10E‐07	43/66	1.53E‐04
4	Mucin type O‐glycan biosynthesis	16/21	1.34E‐07	13/21	0.041
5	Metabolic pathways	302/1118	2.99E‐06	616/1115	2.71E‐24
6	Hypertrophic cardiomyopathy (HCM)	30/64	5.44E‐06	46/64	4.46E‐07
7	Neuroactive ligand‐receptor interaction	44/109	1.82E‐05	72/116	8.60E‐08
8	Focal adhesion	61/173	2.63E‐05	87/173	0.02
9	Biosynthesis of amino acids	26/57	4.11E‐05	38/57	6.88E‐05
10	Arginine and proline metabolism	20/40	6.33E‐05	31/40	2.47E‐06
11	Small cell lung cancer	31/86	7.20E‐05	47/86	3.61E‐04
12	Drug metabolism—cytochrome P450	19/38	9.63E‐05	29/38	9.03E‐06
13	Dilated cardiomyopathy (DCM)	31/69	1.31E‐04	50/69	7.08E‐06
14	Arrhythmogenic right ventricular cardiomyopathy (ARVC)	26/59	1.34E‐04	40/59	1.83E‐04
15	Retinol metabolism	16/32	3.41E‐04	21/33	0.007
16	Pathways in cancer	122/435	6.32E‐04	206/441	0.006
17	Calcium signaling pathway	46/126	6.62E‐04	78/132	9.22E‐05
18	DNA replication	16/34	8.05E‐04	21/34	0.011
19	Glutathione metabolism	20/47	9.36E‐04	31/46	2.37E‐04
20	Metabolism of xenobiotics by cytochrome P450	16/36	0.002	24/37	0.003
21	Alanine, aspartate and glutamate metabolism	13/27	0.002	17/27	0.017
22	Glycolysis/gluconeogenesis	21/53	0.002	33/53	0.001
23	Purine metabolism	35/103	0.002	64/103	9.43E‐06
24	Drug metabolism—other enzymes	20/50	0.002	29/51	0.015
25	beta‐Alanine metabolism	13/28	0.003	22/28	5.23E‐05
26	Chemical carcinogenesis	17/41	0.003	27/43	0.003
27	Regulation of actin cytoskeleton	51/175	0.004	94/177	0.002
28	Pyrimidine metabolism	18/46	0.005	26/46	0.022
29	Hematopoietic cell lineage	20/54	0.007	36/55	1.88E‐04
30	Mineral absorption	16/39	0.007	24/39	0.038
31	Histidine metabolism	9/18	0.007	13/18	0.007
32	Malaria	16/35	0.008	23/35	0.003
33	Glycine, serine and threonine metabolism	13/31	0.008	20/31	0.006
34	Adrenergic signaling in cardiomyocytes	38/117	0.009	64/117	0.005
35	Salivary secretion	23/66	0.01	45/66	6.48E‐05
36	Arachidonic acid metabolism	18/49	0.011	27/49	0.03
37	Cysteine and methionine metabolism	15/39	0.012	25/40	0.005
38	cAMP signaling pathway	45/143	0.013	79/147	0.003
39	Cell adhesion molecules (CAMs)	28/90	0.021	47/91	0.023
40	Pyruvate metabolism	12/32	0.029	24/32	8.92E‐05
41	Influenza A	39/123	0.03	63/121	0.02
42	Steroid hormone biosynthesis	9/22	0.031	17/24	0.003
43	Tryptophan metabolism	11/29	0.032	20/28	0.001
44	Type I diabetes mellitus	8/26	0.033	14/25	0.038
45	Insulin secretion	22/63	0.045	39/63	0.004
46	Cell cycle	34/114	0.05	62/114	0.003

Abbreviations: ALL, total proteins in each pathway; DE, diffentially‐expressed proteins.

*p* < 0.05 values are shown in order of decreasing value.

### 3.4 Ppp6c deletion activates ERK‐ELK1‐FOS and PI3K‐AKT‐CDK/cyclin pathways in the tongue of K and KP mice

**FIGURE 6:**
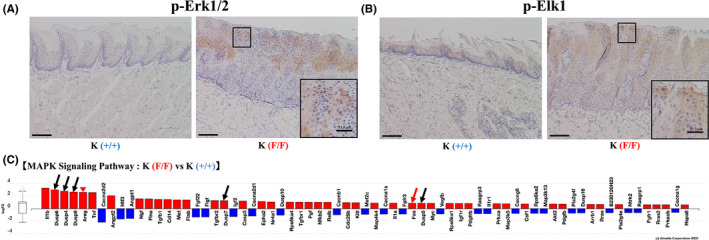
*Ppp6c* deletion activates the ERK‐ELK, AKT‐4EBP1, and CDK/cyclin axes in *the* 4HT‐treated tongue tissue of K mice. Activation of the ERK‐ELK axis. (A, B) Microscopic analysis of the 4HT‐treated tongue of K(F/F) and K(+/+) mice, from tissues shown in Figure 2C. Immunohistochemistry was performed using anti‐phospho ERK1/2 (A) and anti‐phospho Elk1 (B) antibodies. Scale bar: 100 μm. The insets with a scale bar in immunohistochemistry images for K(F/F) tongues is enlarged (×3) one corresponding to the squares in the main image panels. Scale bar: 33.3 μm. (C) Transcripts of components of the MAPK signaling pathway (KEGG 4010) are affected by *Ppp6c* deletion in the tongue tissue of 4HT‐treated K‐mice. mRNA was extracted from tongue tissues of 4HT‐treated K(F/F) and K(+/+) mice, and RNA‐seq performed as described in Methods. Figure was generated using iPathwayGuide (Advaita Bioinformatics) software. Log FC: log of fold‐change in gene expression. Box and whisker plot: box ends are upper and lower quartiles and the span represents the interquartile range. Horizontal line inside the box is the median, and whiskers indicate highest and lowest observations. The level of intensity corresponds to the level of upregulation (red) or downregulation (blue) of the differential genes in the 4HT‐treated K(F/F) tongue versus those of K(+/+). Black arrows indicate negative regulators of the ERK pathway. Red arrow indicates *Fos*, a target of Elk1. Red arrowhead indicates *Areg* (*Amphiregulin*).

ERK and AKT are major RAS effectors governing cell proliferation and growth.^3^ Thus, we screened for proteins that show increased phosphorylation in tongue epithelia of K(F/F) relative to K(+/+) mice on day 13 after 4HT administration using a phosphoprotein antibody array (Table [Link], [Link]). That analysis indicated significant phosphorylation of RAS/MAPK pathway proteins ([Link], [Link]), with increased levels of phosphorylation of MEK1 (T286, T291, S298), ERK1/2 (T202), Elk1(S389), and p90^RSK^ (T359/S363, T573) seen in 4HT‐treated K(F/F) tongue tissue relative to that seen in K(+/+) animals. Immunohistochemistry analysis showed strong staining for phosphorylated ERK1/2 (T202/Y204) throughout the epithelium and phosphorylated Elk1 (S389) primarily in the nucleus in 4HT‐treated K(F/F) tongue, while staining for both was weak in tongue tissues from K(+/+) mice (Figure 6A,B). Figure 6C compares the expression of MAPK signaling factors expressed in 4HT‐treated K(F/F) versus the K(+/+) tongue and indicates a significant increase in expression of MKP factors (*Dusp6*, *Dusp4*, *Dusp9*, *Dusp7*, and *Dusp5*), which function in negative feed‐back to block activated ERK signaling.^22^ We also observed an increase in *Fos*, a target of Elk1^23^ (Figure 6C). In addition, the expression of an EGFR ligand, *Areg* (*Amphiregulin*), was also elevated (Figure 6C). Finally, in KP mice, the effect of *Ppp6c* deletion on the phosphorylation of ERK and Elk1 (Figure S2A) was comparable to effects seen in K mice (Figure 6).

**FIGURE 7:**
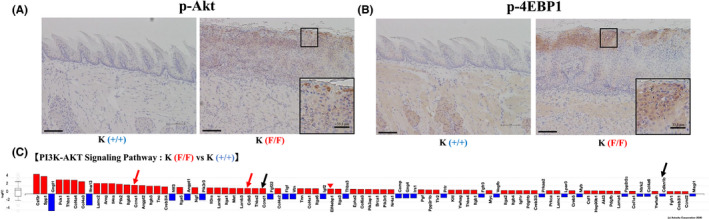
Activation of the AKT‐4EBP1 and CDK/cyclin axes. (A, B) Microscopic analysis of tongue 4HT‐treated tongue of K(F/F) (upper) and K(+/+) mice, as shown in Figure 2C. Immunohistochemistry was performed using anti‐phospho AKT (A) and anti‐phospho 4EBP1 (B) antibodies. Scale bar: 100 μm. The insets were as described in Figure 6A,B. (C) Transcripts of components of the PI3K‐AKT signaling pathways (KEGG 04151) are regulated by *Ppp6c* deletion in the 4HT‐treated tongue of K mice. RNA purification, RNA‐seq, and figure preparation were as described in Figure 6C. Black Arrows indicate *Ccnd1*(*Cyclin D1*) and *Cdkn1b* (*p27*), upregulated and downregulated, respectively, by AKT activation through FOXO.^23^ Red arrows indicate *Ccne1*(*Cyclin E1*) and *Cdk6*, components of CDK/Cyclin activated by AKT. Red arrowhead indicates *Eif4ebp1*(*4EBP1*) a substrate of AKT

Relevant to the PI3K‐AKT pathway, antibody array analysis of 4HT‐treated K(F/F) mice showed the hyperphosphorylation of PDK1(S241), AKT1(T72, S124, T308, Y326, S473), TSC(S939), mTOR(S2481), and 4EBP1(S65) (Table S1B). Thus, we examined phosphorylation levels of AKT1 (T308) and 4EBP1(S65) in 4HT‐treated K(F/F) and K (+/+) mice (Figure 7). AKT1 and 4EBP1 were highly phosphorylated in 4HT‐treated K(F/F) relative to K(+/+) tongue (Figure 7A,B). It is of note that expression of Eif4ebp1 (4EBP1) itself was also upregulated in K(F/F) tongue (Figure 7C). These suggest protein synthesis in the K(F/F) tongue was potentially enhanced.^24^

Figure 7C also indicated an increase in *Ccnd1*(*CyclinD1*) and a decrease in *Cdkn1b* (*p27Kip1*) in 4HT‐treated K(F/F) tongue, expression of both is reportedly regulated by AKT via FOXO.^24^ We also found the upregulation of *Ccne1*(*CyclinE1*) and *Cdk6*, both part of the CDK complex which is activated by AKT through the phosphorylation/inhibition of p21 and p27^24^ (Figure 7C). These data suggest that AKT signaling is up‐regulated and the cell cycle is likely accelerated in 4HT‐treated K(F/F) relative to K(+/+) tongue tissues. Again, effects on the phosphorylation of AKT and 4EBP1 seen following *Ppp6c* deletion in KP mice (Figure S2B) were comparable to those seen in K mice (Figure 7).

### 3.5 Ppp6c deficiency promotes the accumulation of DNA damage and NFκB pathway activation in the tongue tissue of K and KP mice

**FIGURE 8:**
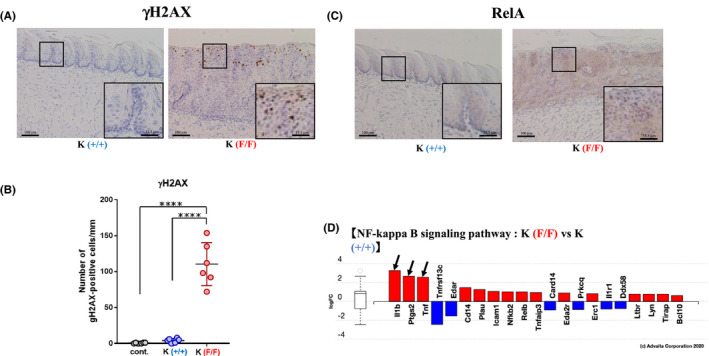
*Ppp6c* deletion promotes the accumulation of DNA damage and NFκB activation in the tongue tissue of K mice. (A) Microscopic analysis of the 4HT‐treated tongue of K(F/F) and K(+/+) mice, as shown in Figure 2C, stained with anti‐γH2AX antibody. Scale bar: 100 μm. The insets with a scale bar in immunohistochemistry images for K(F/F) & K(+/+) tongues are enlarged (×3) one corresponding to the squares in the main image panels. Scale bar: 33.3 μm. (B) Quantification of the number of γH2AX‐positive cells in an area ~1 mm wide in tongue epithelium, counted using samples shown in (A). Data are means derived from six areas in independent samples ±SE. ****p < 0.0001. (C) Microscopic analysis as described in (a) stained with an anti‐RelA antibody. Scale bar: 100 μm. The insets were as described in (A). (D) Expression of genes functioning in NF‐kappa B signaling pathway (KEGG 4064) in the 4HT‐treated tongue of K(F/F) relative to expression seen in K(+/+) mice. mRNA was extracted and figure was generated as described in Figure 6C. Arrows indicate targets of RelA

We next assessed the effects of *Ppp6c* deficiency on DNA repair, as PP6 reportedly functions in DNA‐PK‐mediated repair of non‐homologous end‐joining (NHEJ).^8^ Antibody array data relevant to phospho‐proteins associated with DNA repair pathways are shown in Table S1C. Levels of phosphorylated DNA‐PK (T2638), Histone H2AX (S139), BRCA1 (S1457, S1524) ATRIP (S68/72), Chk1 (S286), and Chk2 (T68, T383) increased in 4HT‐treated K(F/F) relative of K(+/+) tongue, suggesting enhanced DNA damage and activation of DNA repair pathways. As confirmation, we performed immunohistochemical analysis and observed significant accumulation of γH2AX in 4HT‐treated K(F/F) tongue, greater than that seen in K(+/+) tissue (Figure 8A,B). KRAS(G12D) reportedly promotes DNA breaks by inducing reactive oxygen species (ROS); however, the effect of KRAS(G12D) on γH2AX positive number in 4HT‐treated K(+/+) tongue compared to those of wild type was marginal (Figure 8B), suggesting that *Ppp6c* deficiency induces significant DNA damage in tongue tissue expressing K‐ras^G12D^. Similar effects of *Ppp6c* deletion were seen when we conducted this analysis in KP mice (data not shown).

Relevant to B signaling, previous studies show that PP6 specifically dephosphorylates IκBε^9^. Our phosphoprotein analysis showed that IκBε (S22) phosphorylation was particularly up‐regulated in 4HT‐treated K(F/F) compared to K(+/+) tongue tissue (Table S1D). Since IκBε phosphorylation promotes its degradation and allows RelA to enter the nucleus.^25^ We undertook the immunohistological analysis of tongue tissues for the presence of RelA (Figure 8C). RelA was much more abundant than in 4HT‐treated K(F/F) compared to K(+/+) tissue, and its localization was primarily nuclear and seen in cells mainly in the upper part of the epithelium (Figure 8C). Figure 8D shows the ratio of gene expression associated with the NFκB pathway in 4HT‐treated K(F/F) versus K(+/+) tongue tissue. It is noteworthy that expression of the RelA targets *IL*‐*1β*, *Ptgs2*(*COX2*) and *TNF* was markedly enhanced in 4HT‐treated K(F/F) tongue relative to K (+/+) controls (Figure 8D). Finally, KP mice showed comparable effects of *Ppp6c* deletion on RelA localization and target gene expression (data not shown).

## 4 DISCUSSION

Here, we examined the effect of *Ppp6c* deficiency on tumorigenesis in mice with tongue‐specific expression of *K*‐*ras^G12D^* (K‐mice) as well as in *K*‐*ras^G12D^* mice deficient in *Trp53* (KP‐mice). Both K and KP mice developed intraepithelial carcinoma of the tongue and had to be killed approximately 2 weeks after the induction of *K*‐*ras^G12D^*, *Trp53*, and *Ppp6c* mutations. Histopathological analyses performed 2 weeks after induction indicated that Ppp6c deficiency more strongly drove K‐rasG12D‐initiated tumorigenesis than did Trp53 deficiency.

Signaling pathways significantly (*p *< 0.05) deregulated by *Ppp6c* deletion in the presence of *K*‐*ras^G12D^* are shown in Table 1. Among them, ‘Pathways in Cancer’ includes intrinsic factors underlying tumorigenesis and associated with: (a) sustained angiogenesis; (b) evasion of apoptosis; and (c) proliferation. As shown in Figure S3, all three activities are upregulated by transcription factors (*c*‐*myc* and *c*‐*fos*), cell cycle regulators (*CDK*/*cyclin* and *E2F*), and *COX2* in different ways but all depend on ERK/AKT/NFκB signaling, which was activated by *Ppp6c* deletion in the K(F/F) tongue. It is noteworthy that DNA replication was upregulated (ranked 18 among KEGG pathways) and that 16 proteins associated with DNA replication were upregulated (Figure S4A). Cell cycle was also activated (ranked 46) as shown in Figure S4B. It is also notable that upregulated DNA replication requires a large supply of nucleotides and proteins. Overall, metabolic changes associated with nucleotides and amino acids (ranked 5) were also characteristic of *Ppp6c* deficiency. Relevant to nucleotides, purine (ranked 23) and pyrimidine (ranked 28) metabolism was upregulated. Relevant to protein, protein digestion and absorption (ranked 2), biosynthesis of amino acids (ranked 9), and several categories related to amino acid metabolism (ranked 10, 21, 25, 31, 33, 38 and 43) were upregulated. Enhanced amino acid metabolism may reflect enhanced protein synthesis driven by activated 4EBP1 (Figure 7B). Figure 2 shows that *K*‐*ras^G12D^*‐initiated squamous cells begin to divide rapidly when *Ppp6c* is deleted and develop into tumor within 2 weeks. Such enhanced proliferation may be enabled by *Ppp6c* deletion in the presence of *K*‐*ras^G12D^*, which supports cell proliferation by facilitating activity of cell machineries required for rapid cell division.

*Ppp6c* deletion likely regulates cytokine–cytokine receptor interaction (ranked 1 in Table 1; Figure S5). As shown in Figure S5, the expression level of several cytokines such as *Ccl3*, *Cxcl3*, *Cxcl1*, *Cxcl5*, *Ccl2* is highly elevated (Figure S5). It is likely due to the enhancement of the TNF signaling pathway regulated by NF*κ*B signaling^26^ (Figure S6A). In 4HT‐K(F/F) tongue, TNF signaling is positively regulated by TNF (Figure S6A) and leading to an explosion of cytokines such as *Cxcl3*, *Cxcl1*, and *Ccl2* but also proinflammatory cytokine genes *TNF*, *IL*‐*1β*, and extracellular remodeling factor such as *Mmp9*, *and Mmp3*, and *COX2* (Figure S6B). These factors together may lead to chronic and excessive inflammatory states. As shown in Figures 3 (HE) and 4 (HE), greater infiltration by inflammatory cells was seen in the stroma of K(F/F) relative to K(+/+) mice. Chronic inflammation reportedly promotes tumor progression in mice and humans,^25^, ^26^, ^27^ and our findings strongly suggest that inflammation driven by *Ppp6c* deficiency in the presence of oncogenic RAS is a factor in tongue carcinoma.

To examine the effects of p53 loss on signaling, we compared the transcriptome of 4HT‐treated KP(F/F) tongue to that of K(F/F) animals (Table S2). ‘The Pathway in Cancer’ is identified in Table S2. We found that sustained angiogenesis, evasion of apoptosis, and proliferation were enhanced in the KP(F/F) relative to the K(F/F) tongue. (Figure S7), indicating that by 13 days, KRAS tumorigenicity caused by Ppp6c deficiency is further enhanced by p53. This finding also suggests that if animals remain viable beyond 2 weeks, 4HT‐treated KP(F/F) tongues may be more malignant than those of K(F/F).

Recently, however, some have proposed a different mechanism for PP6 protein downregulation. Fujiwara et al. showed that PP6 associates with the autophagic adaptor protein p62/SQSTM1 and is degraded in a p62‐dependent manner.^28^ Nonetheless, whatever the mechanism, our results strongly suggest that in the presence of oncogenic RAS, PP6 downregulation promotes Ras‐initiated tumorigenesis.

Numerous anti‐Ras reagents have been proposed as a treatment for RAS‐initiated cancers, but successful therapies remain elusive. Here, we found that PP6 functions as a tumor suppressor by suppressing the activity of ERK, AKT, and NFκB. We conclude that PP6 activators could provide a novel therapeutic strategy to repress cancer pathways and cytokine–cytokine receptor interactions.

## CONFLICT OF INTEREST

The authors declare no conflict of interest.

## Supporting information

Figure S1.

Figure S2.

Figure S3.

Figure S4.

Figure S5.

Figure S6.

Figure S7.

Figure S8.

Figure S9.

Table S1.

Table S2.

Supinfo.

## Data Availability

The data that support the findings of this study are available from the corresponding author upon reasonable request.
