# Supplementary figures and images for: Ppp6c deficiency accelerates K‐ras G12D ‐induced tongue carcinogenesis
Source: Cancer Med. 2021 Jun 18;10(13):4451–64. doi: 10.1002/cam4.3962 (PMC8267137; doi:10.1002/cam4.3962)

Fig. S1  
A

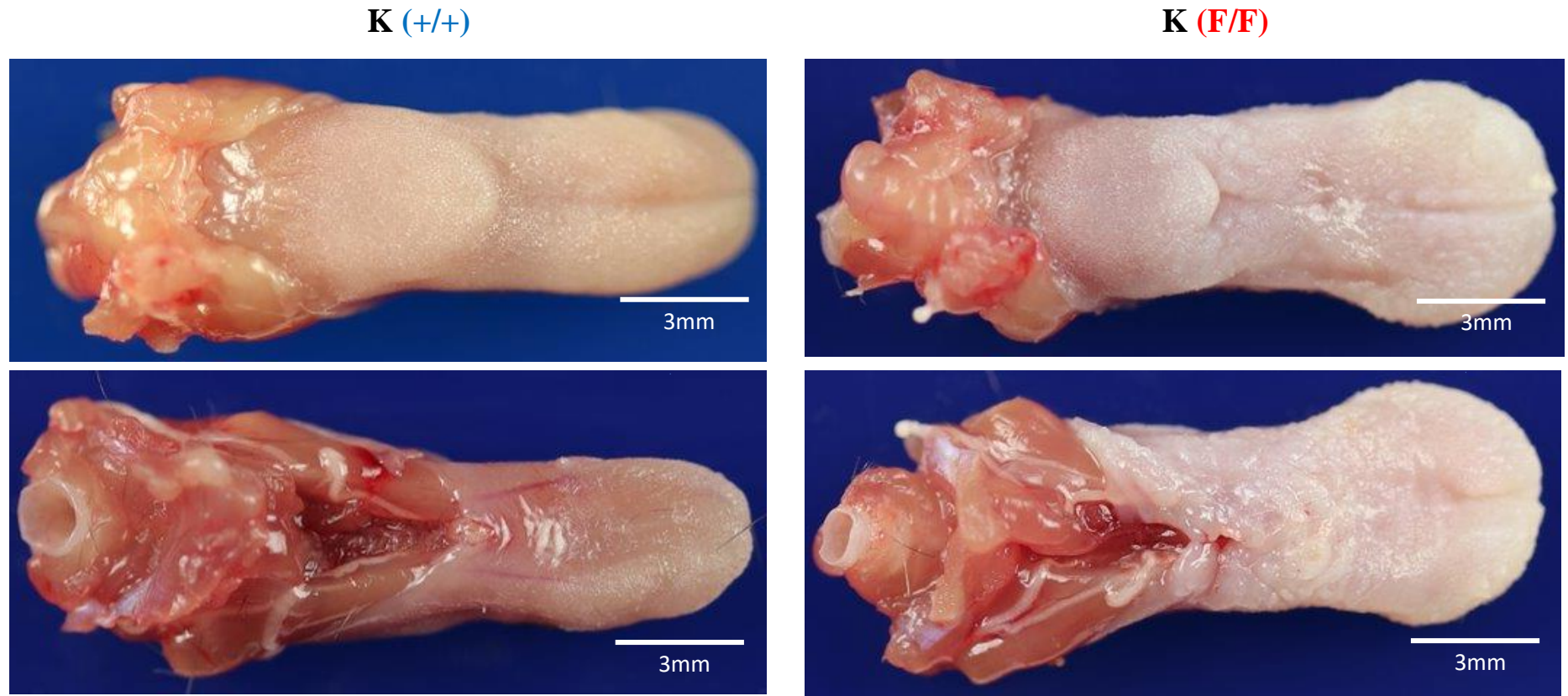

**Fig. S1** Macroscopic views of representative 4HT-treated tongues of K and KP mice.

Supplement: Supplementary file 1 — Figure S1. [file CAM4-10-4451-s001.pdf]

Fig. S1  
B

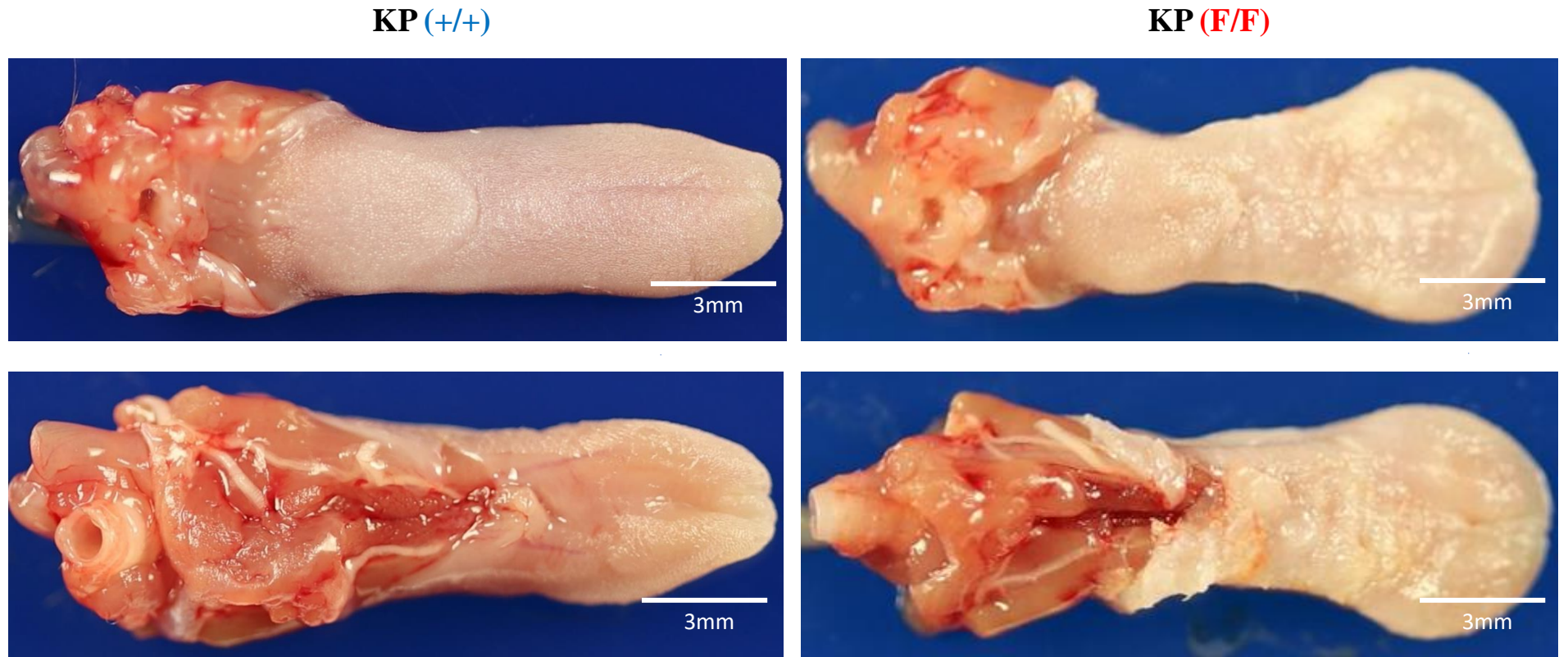

**Fig. S1** Macroscopic views of representative 4HT-treated tongues of K and KP mice.

Supplement: Supplementary file 2 — Figure S2. [file CAM4-10-4451-s005.pdf]
