# Supplementary material for: Ppp6c deficiency accelerates K‐ras G12D ‐induced tongue carcinogenesis
Source: Cancer Med. 2021 Jun 18;10(13):4451–64. doi: 10.1002/cam4.3962 (PMC8267137; doi:10.1002/cam4.3962)
Supplement: Supplementary file 3 — Figure S3. [file CAM4-10-4451-s002.pdf]

Fig. S2

A

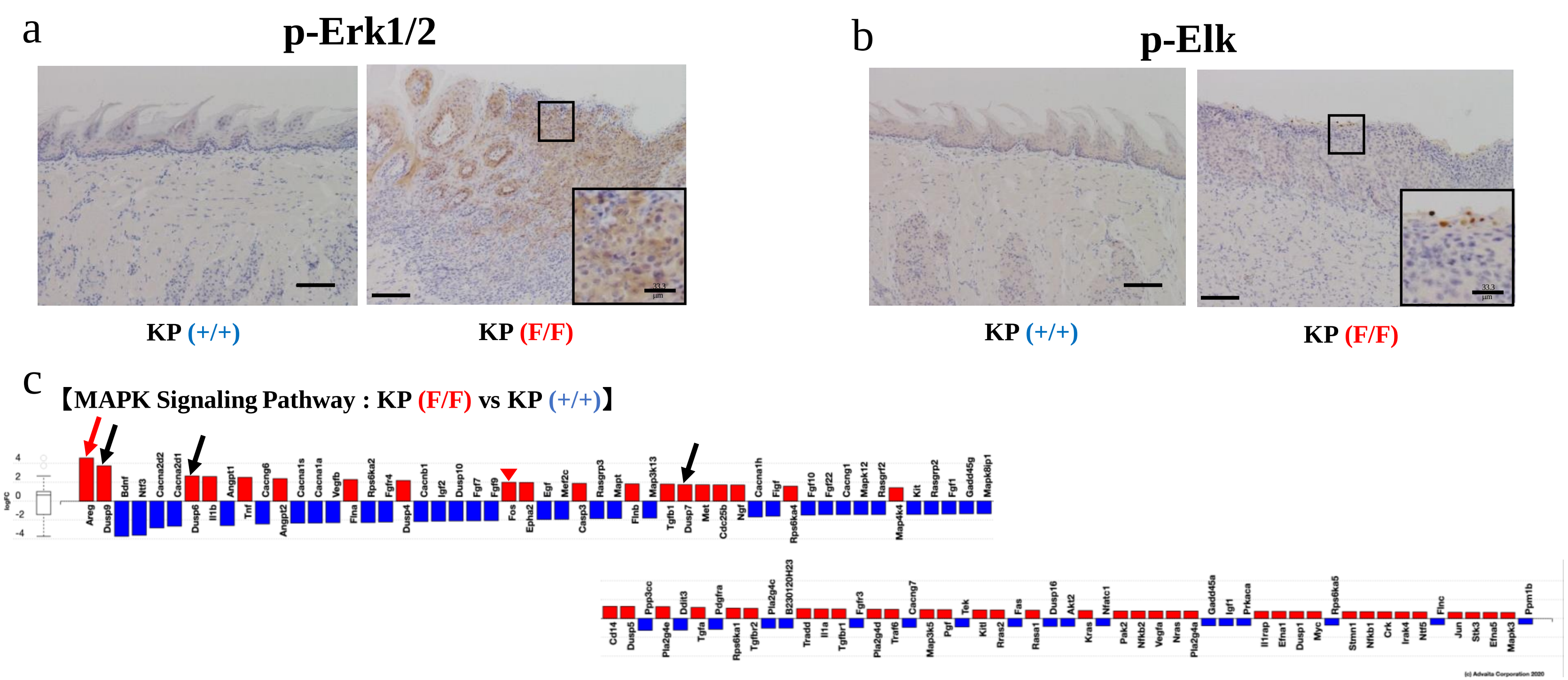

Fig. S2 *Ppp6c* deletion activates MAPK and PI3-AKT signaling in tongue of KP mice
A: Activation of the ERK-ELK axis.

a,b: Microscopic analysis of 4HT-treated tongues of KP(F/F) and KP(+/+) mice, from tissues shown in Fig. 1Bf. Immunohistochemistry was performed using anti-phospho ERK1/2 (a) and anti-phospho Elk1(b) antibodies. Scale bar: 100 μm. Insets with a scale bar in immunohistochemistry images for KP(F/F) tongues are enlarged (x3) and correspond to squares in main images. Scale bar: 33.3 μm.

c: Levels of transcripts encoding MAPK pathway factors (KEGG 4010) change after *Ppp6c* deletion in tongue of 4HT-treated KP-mice. mRNA was extracted from tongue tissues and RNA-seq performed as in described in Methods. Figure was generated using iPathwayGuide (Advaita Bioinformatics) software. log FC: log fold-change in gene expression. Box and whisker plot: box ends are upper and lower quartiles and the span represents the interquartile range. Horizontal line inside box is the median, and whiskers indicate highest and lowest observations. Black arrows indicate negative regulators of the ERK pathway. Red arrowhead indicates *Fos*, a target of Elk1. Red arrow indicates *Areg*（Amphiregulin）.
