# Supplementary material for: Ppp6c deficiency accelerates K‐ras G12D ‐induced tongue carcinogenesis
Source: Cancer Med. 2021 Jun 18;10(13):4451–64. doi: 10.1002/cam4.3962 (PMC8267137; doi:10.1002/cam4.3962)
Supplement: Supplementary file 4 — Figure S4. [file CAM4-10-4451-s006.pdf]

Fig. S2

B

a p-Akt

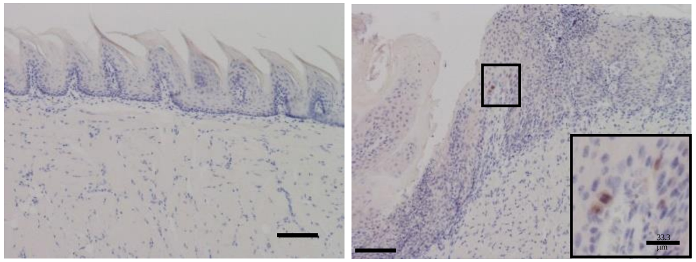

KP (+/+) KP (F/F)

b p-4EBP1

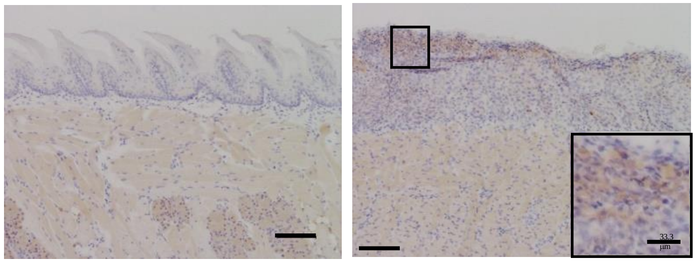

KP (+/+) KP (F/F)

c 【PI3-AKT Pathway : KP (F/F) vs KP (+/+)】

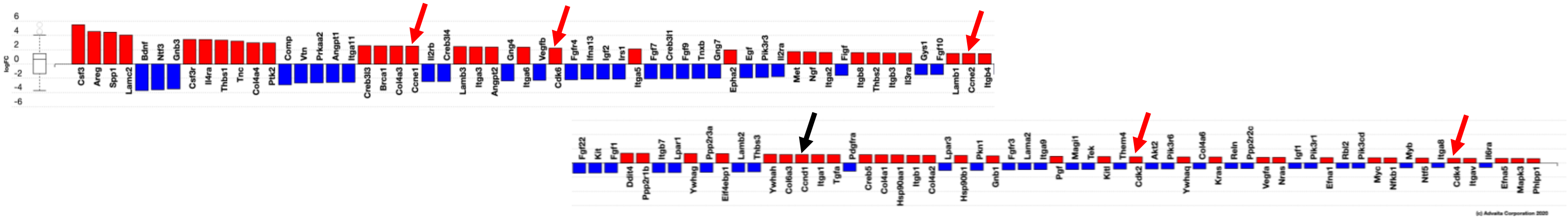

Fig. S2 *Ppp6c* deletion activates MAPK and PI3-AKT signaling in tongue of KP mice

B: Activation of the AKT-4EBP1 and CDK/cyclin axes.

a, b: Microscopic analysis of 4HT-treated tongue of KP(F/F) and KP(+/+) mice, as shown in Fig. 1Bf. Immunohistochemistry was performed using anti-phospho AKT(a) and anti-phospho 4EBP1(b) antibodies. Scale bar: 100 µm. Insets are as described in Fig. S2A.

c: Transcripts encoding PI3K-AKT signaling factors (KEGG 04151) are regulated by *Ppp6c* deletion in 4HT-treated tongue of KP mice. RNA purification, RNA-seq, and figure preparation are described in Methods. Black arrow indicates *Ccnd1*(*Cyclin D1*) . Red arrows indicate *Ccne1*(*Cyclin E1*), *Ccne2*(*Cyclin E2*), *cdk2*, *cdk4*, and *cdk6*, which are components of the CDK/Cyclin pathway activated by AKT.
