# Supplementary material for: Ppp6c deficiency accelerates K‐ras G12D ‐induced tongue carcinogenesis
Source: Cancer Med. 2021 Jun 18;10(13):4451–64. doi: 10.1002/cam4.3962 (PMC8267137; doi:10.1002/cam4.3962)
Supplement: Supplementary file 5 — Figure S5. [file CAM4-10-4451-s003.pdf]

Fig. S3

【Pathways in Cancer : K (F/F) vs K (+/+)】

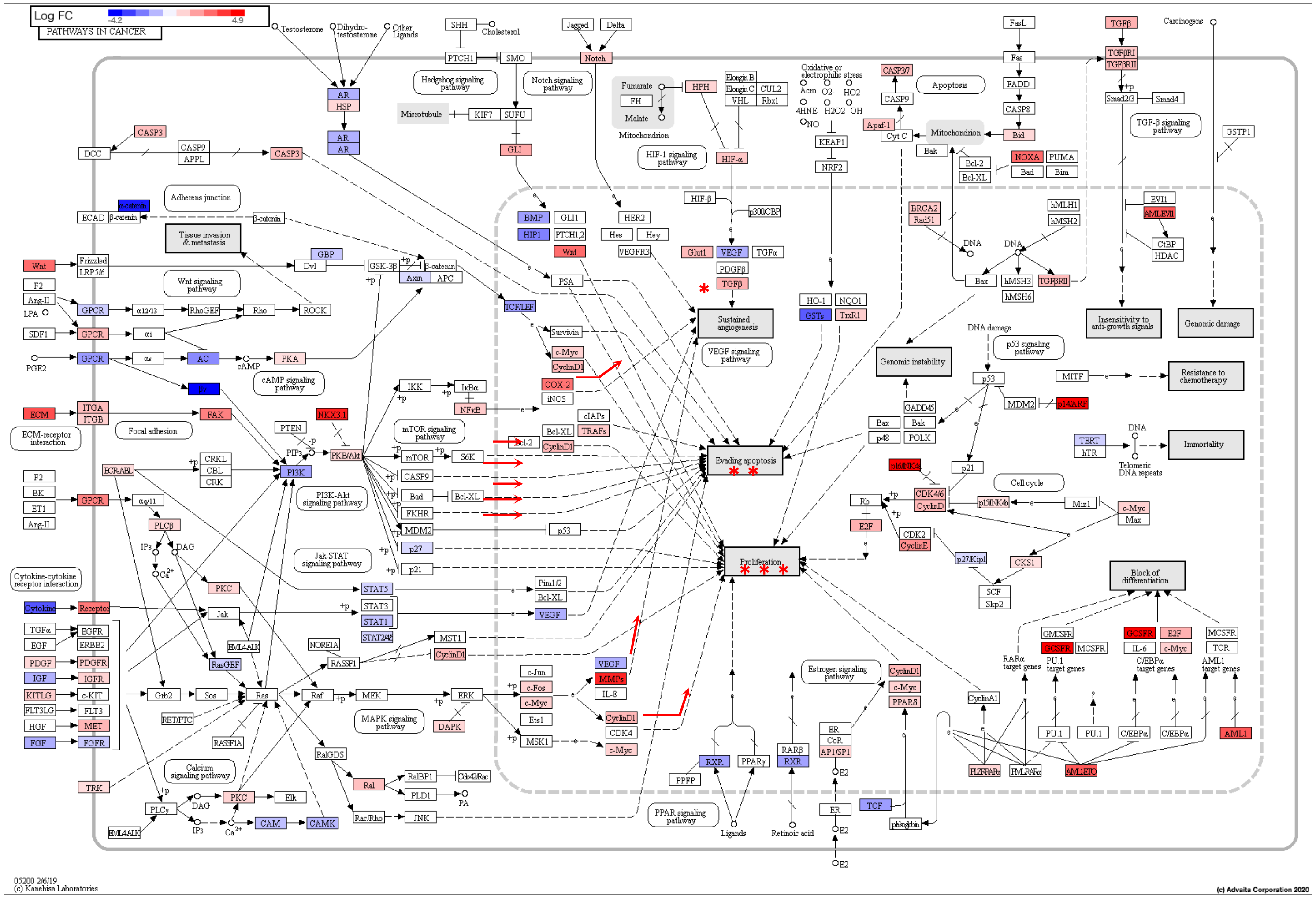

Fig. S3 ***Ppp6c*** **deletion promotes Pathways in Cancer in tongue tissue of K mice**

Shown is schematic of the KEGG 05200 pathway “Pathways in Cancer” combined with gene expression data from this study. Gene products are indicated with small rectangles and arrows indicate molecular interactions (see https://www.genome.jp/kegg/document/help_pathway.html for detailed notations). Up- and down-regulated genes in K(F/F) relative to K(+/+) tumors are indicated in red and blue, respectively. Color shading represents the extent of up- or down-regulation. Gray rectangles with thick lines indicate specific pathways in cancer. For the box “Sustained angiogenesis” (*), “Evading apoptosis” (**) and “Proliferation” (***), the arrays from ERK/AKT/NFκB signaling are converging.
