# Supplementary material for: Ppp6c deficiency accelerates K‐ras G12D ‐induced tongue carcinogenesis
Source: Cancer Med. 2021 Jun 18;10(13):4451–64. doi: 10.1002/cam4.3962 (PMC8267137; doi:10.1002/cam4.3962)
Supplement: Supplementary file 6 — Figure S6. [file CAM4-10-4451-s007.pdf]

Fig. S4

A

【 DNA replication : K (F/F) vs K (+/+)】

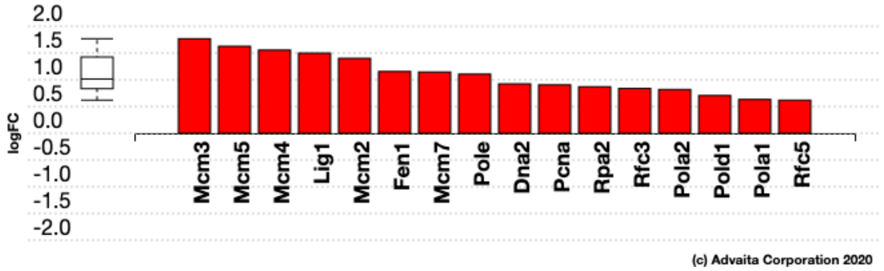

B

【 Cell Cycle : K (F/F) vs K (+/+)】

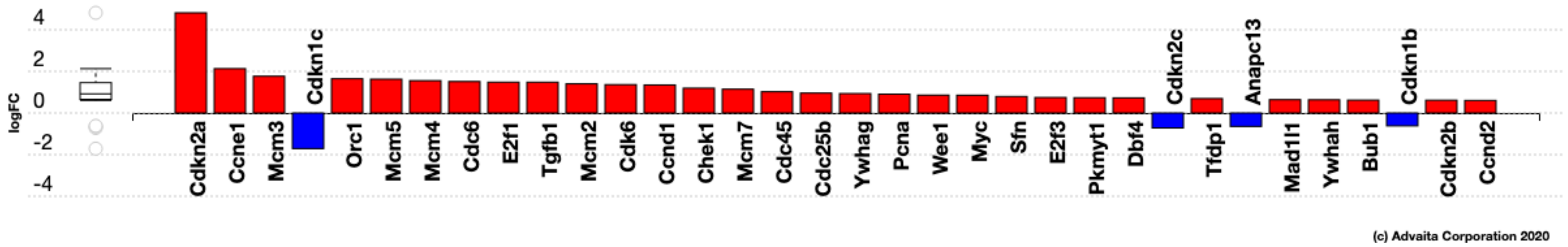

Fig. S4 ***Ppp6c*** **deletion activates DNA replication and Cell cycle pathways in tongue of K mice**

A : Upregulation of genes involved in the DNA replication pathway (KEGG 3030) following *Ppp6c* deletion in tongue of 4HT-treated K-mice. mRNA was extracted from tongue tissues and RNA-seq was performed as described in Methods. The figure was generated using iPathwayGuide (Advaita Bioinformatics) software. log FC: log fold-change in gene expression. Box and whisker plot: box ends are upper and lower quartiles and the span represents the interquartile range. Horizontal line inside the box is the median, and whiskers indicate highest and lowest observations.

B : Expression of genes functioning in the Cell cycle pathway (KEGG 4110) in 4HT-treated tongue of K(F/F) relative to K(+/+) mice. mRNA was extracted and figure was generated as described in the Methods.
