# Supplementary material for: Ppp6c deficiency accelerates K‐ras G12D ‐induced tongue carcinogenesis
Source: Cancer Med. 2021 Jun 18;10(13):4451–64. doi: 10.1002/cam4.3962 (PMC8267137; doi:10.1002/cam4.3962)
Supplement: Supplementary file 7 — Figure S7. [file CAM4-10-4451-s011.pdf]

Fig. S5

【Chemokine-cytokine receptor interaction : K (F/F) vs K (+/+)】

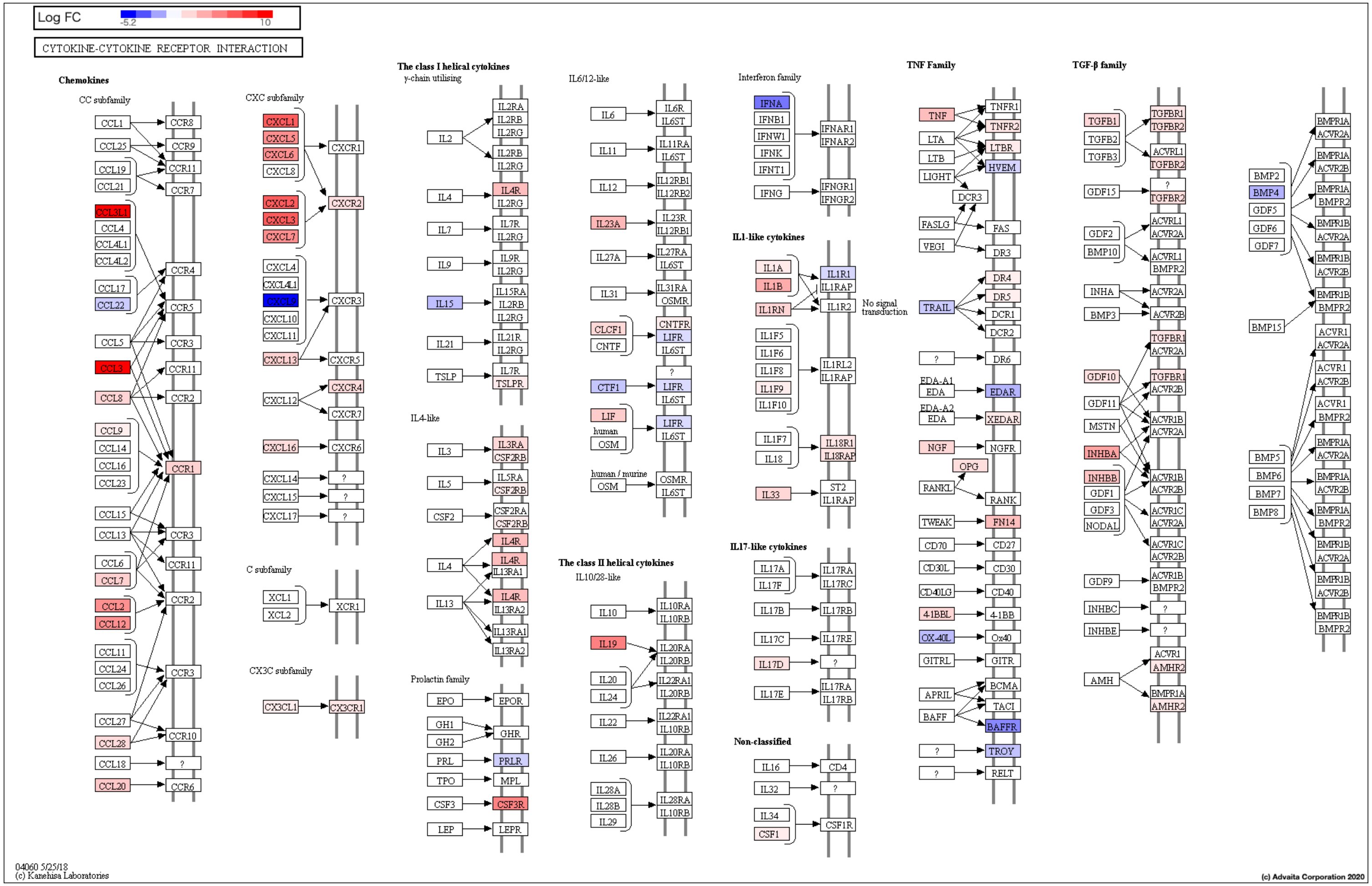

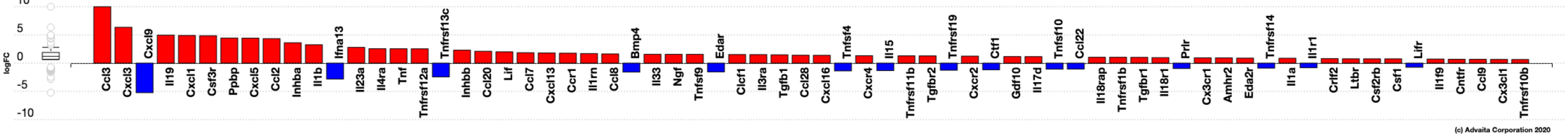

Fig. S5 *Ppp6c* deletion activate Cytokine-cytokine receptor interaction in tongue tissue of K mice

Expression of genes functioning in Cytokine-cytokine receptor interaction pathway (KEGG 04668) in 4HT-treated tongue of K(F/F) relative to expression seen in K(+/+) mice. mRNA was extracted and figure was generated as described in the Methods.
