# Supplementary material for: Ppp6c deficiency accelerates K‐ras G12D ‐induced tongue carcinogenesis
Source: Cancer Med. 2021 Jun 18;10(13):4451–64. doi: 10.1002/cam4.3962 (PMC8267137; doi:10.1002/cam4.3962)
Supplement: Supplementary file 9 — Figure S9. [file CAM4-10-4451-s008.pdf]

Fig. S7

【Pathways in Cancer : KP (F/F) vs K (F/F) 】

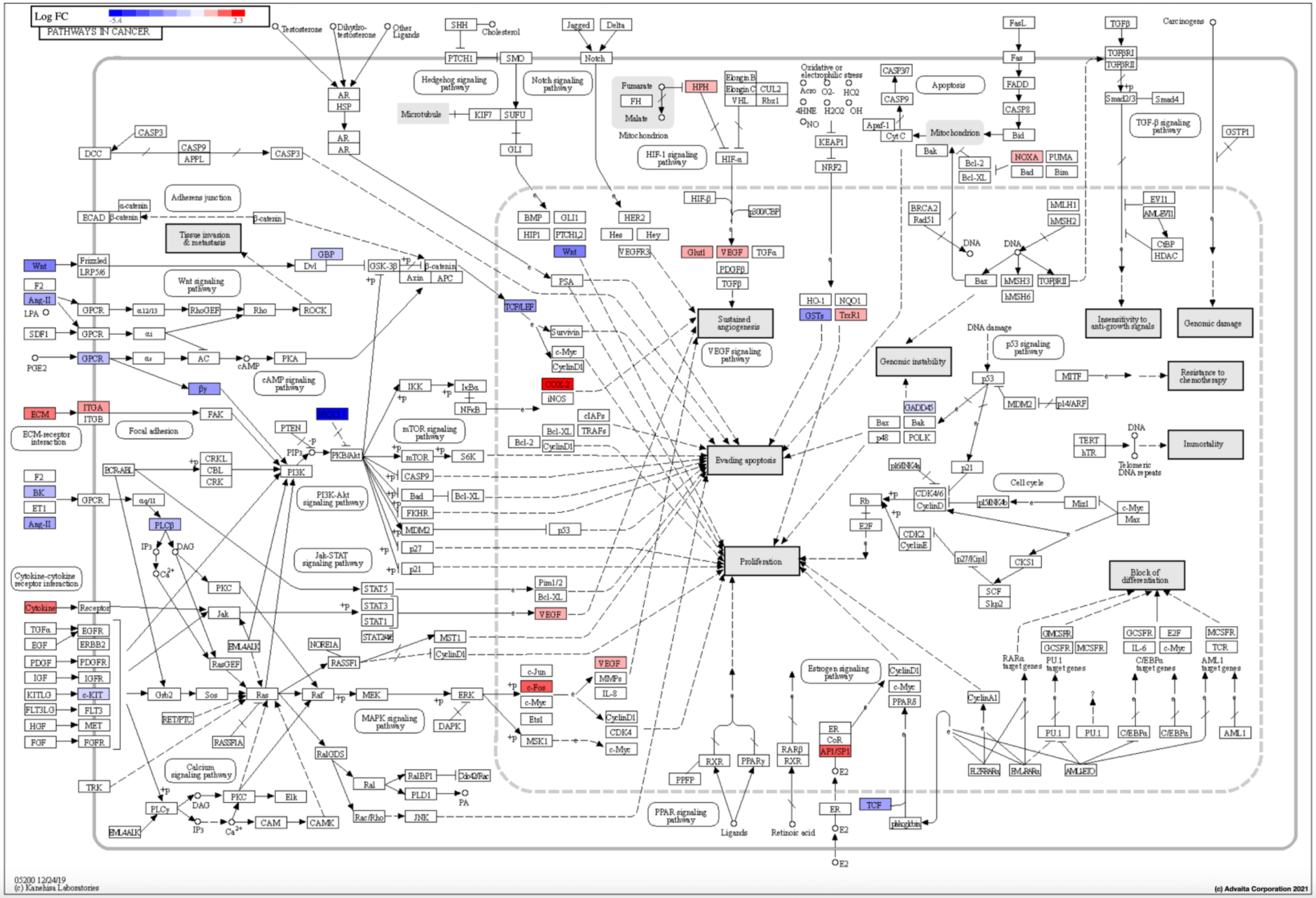

Fig. S7 *Trp53* deletion promotes Pathways in Cancer in tongue tissue of K(F/F) mice

This panel is the schematic diagram of KEGG 05200 pathway “Pathways in Cancer” with gene expression data of the present study. The gene products are indicated with small rectangles and the arrows indicate the molecular interactions (see https://www.genome.jp/kegg/document/help_pathway.html for detailed notations). The upregulated and downregulated genes in KP(F/F) tumors comparing with K(F/F) tumors were indicated with red and blue, respectively. The contrast of the colors represents the extent of up or down regulations of the genes. Gray rectangles with thick lines indicate specific pathways in cancer and the three pathways, “Sustained angiogenesis”, “Evading apoptosis” and “Proliferation” show accumulation of the arrows from the up or downregulated genes in this study.
