# Supplementary material for: Ppp6c deficiency accelerates K‐ras G12D ‐induced tongue carcinogenesis
Source: Cancer Med. 2021 Jun 18;10(13):4451–64. doi: 10.1002/cam4.3962 (PMC8267137; doi:10.1002/cam4.3962)
Supplement: Supplementary file 10 — Table S1. [file CAM4-10-4451-s009.pdf]

Table S1 Phosphorylation levels of proteins in various signaling pathways

**a. RAS/MAPK**	
MEK1 (Phospho-Thr286)	1.997
MEK1 (Phospho-Thr291)	10.976
MEK1 (Phospho-Ser298)	2.352
ERK1/2 (Phospho-Thr202)	1.277
Elk1 (Phospho-Ser389)	9.579
Elk1 (Phospho-Thr417)	1.108
p90RSK (Phospho-Thr359/Ser363)	8.870
p90RSK (Phospho-Thr573)	1.609
**b. PI3K/Akt/mTOR**	
PI3-kinase p85-subunit alpha/gamma (Phospho-Tyr467/Tyr199)	1.155
PTEN (Phospho-Ser370)	7.920
PTEN (Phospho-Ser380/Thr382/Thr383)	6.562
PDK1 (Phospho-Ser241)	8.324
AKT1 (Phospho-Thr72)	2.358
AKT1 (Phospho-Ser124)	2.034
AKT1 (Phospho-Thr308)	8.166
AKT1 (Phospho-Tyr326)	3.237
AKT1 (Phospho-Ser473)	1.443
Tuberin/TSC2 (Phospho-Ser939)	12.574
Tuberin/TSC2 (Phospho-Thr1462)	0.969
mTOR (Phospho-Ser2481)	2.491
4E-BP1 (Phospho-Ser65)	4.090
p70S6K (Phospho-Ser371)	1.069
p70S6K (Phospho-Ser418)	0.950
p70S6K (Phospho-Thr421)	1.047
**c. DNA repair**	
DNA-PK (Phospho-Thr2638)	5.822
Histone H2A.X (Phospho-Ser139)	8.671
BRCA1 (Phospho-Ser1457)	2.038
BRCA1 (Phospho-Ser1524)	7.575
ATRIP (Phospho-Ser68/72)	22.584
Chk1 (Phospho-Ser280)	1.046
Chk1 (Phospho-Ser286)	12.743
Chk2 (Phospho-Thr68)	6.706
Chk2 (Phospho-Thr383)	5.574
**d. NF-κB**	
IkB-alpha (Phospho-Ser32/36)	0.088
IkB-alpha (Phospho-Tyr42)	1.526
IkB-beta (Phospho-Thr19)	1.633
IkB-epsilon (Phospho-Ser22)	8.327

The degree of phosphorylation of proteins in the 4HT-treated K(F/F) tongue is expressed as a ratio of that seen in 4HT-treated K(+/+) tongue, with the latter value set to 1. One sample of each genotype was analyzed by using Phosphoprotein microarray analyses.
