# Supplementary material for: Ppp6c deficiency accelerates K‐ras G12D ‐induced tongue carcinogenesis
Source: Cancer Med. 2021 Jun 18;10(13):4451–64. doi: 10.1002/cam4.3962 (PMC8267137; doi:10.1002/cam4.3962)
Supplement: Supplementary file 11 — Table S2. [file CAM4-10-4451-s004.pdf]

Table S2 Signaling pathway analysis in KP(F/F) versus K(F/F) mice.

Rank	KEGG-pathway	# genes (DE/ALL)	*p* -value
**1**	Neuroactive ligand-receptor interaction	17 / 107	2.67E-06
**2**	Drug metabolism - cytochrome P450	11 / 38	7.75E-06
**3**	ECM-receptor interaction	16 / 63	1.36E-05
**4**	Amoebiasis	14 / 77	4.55E-05
**5**	Renin-angiotensin system	7 / 19	5.80E-05
**6**	Staphylococcus aureus infection	11 / 56	7.14E-05
**7**	Protein digestion and absorption	12 / 63	8.66E-05
**8**	Salivary secretion	12 / 63	1.56E-04
**9**	Retinol metabolism	8 / 31	2.25E-04
**10**	Estrogen signaling pathway	15 / 110	3.00E-04
**11**	Chemical carcinogenesis	9 / 43	4.18E-04
**12**	PI3K-Akt signaling pathway	25 / 267	4.76E-04
**13**	PPAR signaling pathway	10 / 54	8.39E-04
**14**	Focal adhesion	18 / 173	3.00E-03
**15**	Pancreatic secretion	8 / 62	3.00E-03
**16**	Metabolism of xenobiotics by cytochrome P450	7 / 38	5.00E-03
**17**	Arachidonic acid metabolism	8 / 49	5.00E-03
**18**	Insulin secretion	9 / 60	5.00E-03
**19**	Linoleic acid metabolism	5 / 20	6.00E-03
**20**	Glutathione metabolism	8 / 51	0.006
**21**	Taste transduction	5 / 29	0.006
**22**	cAMP signaling pathway	14 / 140	0.007
**23**	Cortisol synthesis and secretion	6 / 44	0.007
**24**	Cytokine-cytokine receptor interaction	15 / 163	0.008
**25**	Nitrogen metabolism	4 / 14	0.009
**26**	Tyrosine metabolism	5 / 23	0.01
**27**	Hematopoietic cell lineage	8 / 56	0.01
**28**	IL-17 signaling pathway	10 / 77	0.011
**29**	Pathways in cancer	31 / 432	0.014
**30**	Complement and coagulation cascades	7 / 43	0.015
**31**	Renin secretion	6 / 52	0.016
**32**	Cocaine addiction	6 / 33	0.017
**33**	cGMP-PKG signaling pathway	13 / 129	0.018
**34**	African trypanosomiasis	5 / 24	0.018
**35**	Metabolic pathways	68 / 1155	0.018
**36**	Glucagon signaling pathway	10 / 88	0.018
**37**	Cysteine and methionine metabolism	6 / 38	0.019
**38**	Relaxin signaling pathway	12 / 111	0.019
**39**	Insulin resistance	10 / 96	0.022
**40**	alpha-Linolenic acid metabolism	4 / 18	0.022
**41**	Regulation of lipolysis in adipocytes	6 / 42	0.023
**42**	Serotonergic synapse	9 / 78	0.025
**43**	Human papillomavirus infection	21 / 275	0.03
**44**	Amphetamine addiction	6 / 52	0.033
**45**	Parathyroid hormone synthesis, secretion and action	9 / 87	0.034
**46**	Mucin type O-glycan biosynthesis	4 / 22	0.041
**47**	Aldosterone synthesis and secretion	7 / 75	0.041
**48**	Transcriptional misregulation in cancer	11 / 144	0.042
**49**	Small cell lung cancer	9 / 86	0.046
**50**	AGE-RAGE signaling pathway in diabetic complications	9 / 94	0.048

DE: Diffentially-expressed proteins. All: Total proteins in each pathway.

$p<0.05$ values are shown in order of decreasing value.
