# Supplementary material for: Ppp6c deficiency accelerates K‐ras G12D ‐induced tongue carcinogenesis
Source: Cancer Med. 2021 Jun 18;10(13):4451–64. doi: 10.1002/cam4.3962 (PMC8267137; doi:10.1002/cam4.3962)
Supplement: Supplementary file 12 — Supinfo. [file CAM4-10-4451-s010.docx]

**Doc S1 Materials and Methods**

***Phosphoprotein microarray analysis***

The Phospho Explorer antibody microarray, which was designed and manufactured by Full Moon Biosystems, Inc. (Sunnyvale, CA), contains 1,318 antibodies was used. The antibody array experiment was performed by Full Moon Biosystems, according to their established protocols. Tongues of K(F/F) and K(+/+) mice were painted with 4HT as described above. At 13 days later, mice were anesthetized, and the surface of the tongue was harvested and frozen. Total protein was extracted from frozen tissue using Protein Extraction Buffer (Full Moon BioSystems Inc.) and transferred to the lab on dry ice. Proteins were labeled with biotin and placed on preblocked microarray slides. Detection of total and phosphorylated proteins was conducted using Cy3-conjugated streptavidin, and phosphorylated protein levels were normalized to those of corresponding total proteins.

***RNA preparation and Sequencing***

Total RNA was extracted from fresh frozen tissue using RNeasy Plus Universal Mini Kit (QIAGEN). mRNA in total RNA was converted to a library of template molecules suitable for subsequent cluster generation using the Illumina TruSeq Stranded mRNA Library Prep Kit. Following purification of poly-A containing mRNA with poly-T oligo-attached magnetic beads, mRNA was fragmented in the presence of divalent cations at 95°C for 5 min. Fragments were copied into first strand cDNA using reverse transcriptase and random primers followed by second strand synthesis using DNA Polymerase I and RNase H. To achieve strand specificity, dUTP was incorporated during second strand synthesis. Fragments then underwent an end repair process via addition of a single ‘A’ base, and adaptor ligation. Products were purified and enriched using PCR to create the final cDNA library. Libraries were sequenced on an Illumina NovaSeq 6000 platform in paired-end 100 bp configuration.

***Transcriptome analysis***

Adapter and low-quality sequences were removed by cutadapt^1^ (v1.2.1). After quality control, poly-A/T sequences were also removed by PRINSEQ^2^ (v0.19.2). For gene expression analysis, trimmed reads were aligned to the reference mouse genome (GRCm38/mm10) using TopHat^3^ (v2.0.13). Mapped reads were assembled by Cufflinks^4^ (v2.2.1), and transcripts across all samples were merged by Cuffmerge, a part of the Cufflinks package. The “fragments per kilo base per million map reads” value (FPKM) was calculated with Cuffquant, and differential expression analysis (calculating fold change and testing the statistical significance) between two groups was performed with Cuffdiff (Cuffquant and Cuffdiff are programs involved in the Cufflinks package). We considered a gene considered differentially-expressed if we observed a significant difference between the two groups (q-value < 0.05). Hierarchical clustering analysis was performed on differentially-expressed genes using the hclust function in R, according to 1 minus Pearson's correlation coefficient and average agglomeration method．Heat map was generated using BioConductor package HeatPlus. The statistical analysis of pathways was carried out using iPathwayGuide^5^ (Advaita Bioinformatics).

**Doc S1 References**

1. Martin M. Cutadapt removes adapter sequences from high-throughput sequencing reads. EMBnet. journal, North America. 2011;9

<http://journal.embnet.org/index.php/embnetjournal/article/view/200/479>

1. Schmieder R, Edwards R. Quality control and preprocessing of metagenomic datasets. Bioinformatics. 2011;27(6):863-4.
2. Trapnell C, Pachter L, Salzberg SL. TopHat: discovering splice junctions with RNA-Seq. Bioinformatics. 2009;25(9):1105-11.
3. Trapnell C, Williams BA, Pertea G, et al. Transcript assembly and quantification by RNA-Seq reveals unannotated transcripts and isoform switching during cell differentiation. Nat Biotechnol. 2010;28(5):511-5.
4. Draghici S, Khatri P, Tarca AL, et al. A systems biology approach for pathway level analysis. Genome Res. 2007;17(10):1537-45.
