# Supplementary material for: Ppp6c deficiency accelerates K‐ras G12D ‐induced tongue carcinogenesis
Source: Cancer Med. 2021 Jun 18;10(13):4451–64. doi: 10.1002/cam4.3962 (PMC8267137; doi:10.1002/cam4.3962)
Supplement: Supplementary file 8 — Figure S8. [file CAM4-10-4451-s012.pdf]

Fig. S6 A 【TNF signaling pathway : K (F/F) vs K (+/+)】

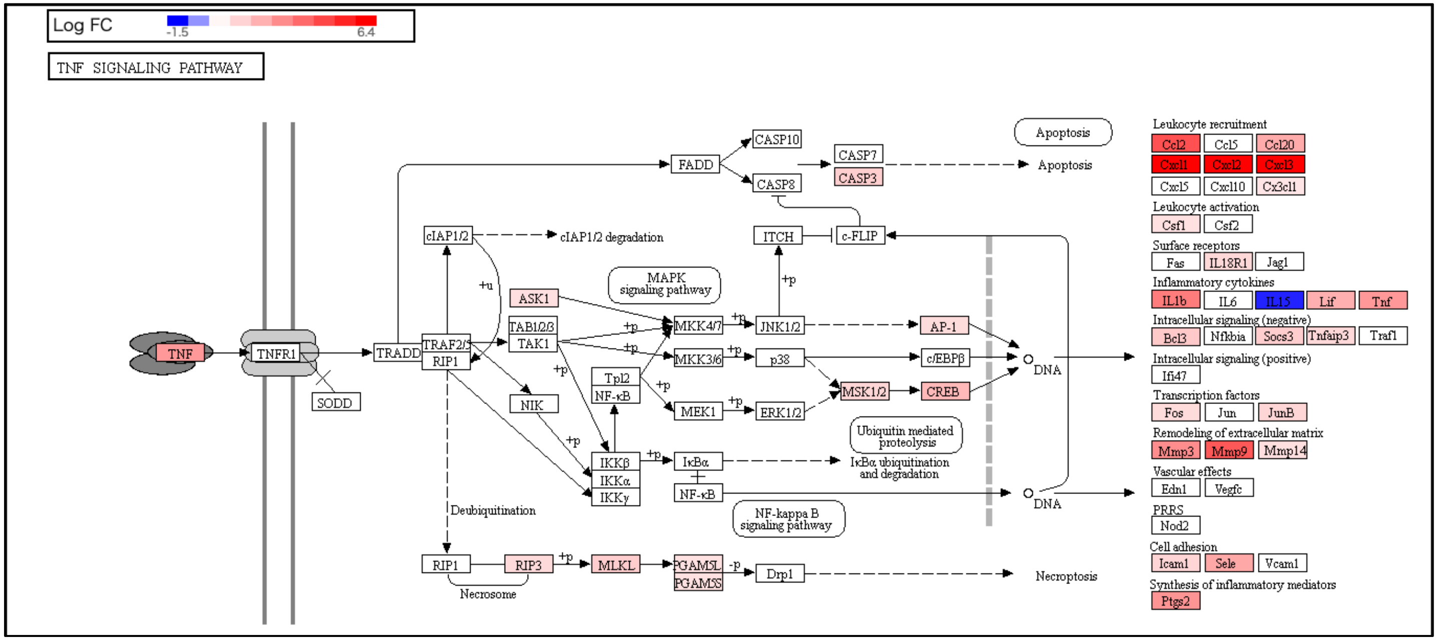

B 【TNF signaling pathway : K (F/F) vs K (+/+)】

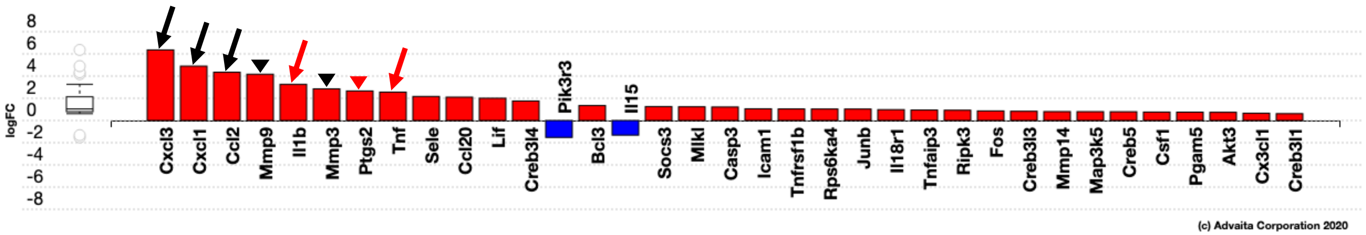

Fig. S6 *Ppp6c* deletion activate TNF signaling pathway in tongue tissue of K mice

A: The schematic diagram of KEGG 04060 pathway “TNF signaling pathway” with gene expression data of the present study. The diagram was generated as described in Fig. S1. The upregulated and downregulated genes in K(F/F) tumors comparing with K(+/+) tumors were indicated with red and blue, respectively.

B: Expression of genes functioning in TNF signaling pathway (KEGG 04060) in 4HT-treated tongue of K(F/F) relative to expression seen in K(+/+) mice. mRNA was extracted and figure was generated as described in Fig. S2A. Black arrows indicate chemotactic cytokines /chemokines (*Cxcl3*, *Cxcl1* and *Ccl2*). Red arrows indicate proinflammatory cytokines (*TNF* and *IL-1β*). Black arrowheads indicate extracellular remodeling factors (*Mmp9*, and *Mmp3*). Red arrowhead indicates *Pags2* (*COX2*).
